# Efficacy of stem cell therapy in animal models of intracerebral hemorrhage: an updated meta-analysis

**DOI:** 10.1186/s13287-022-03158-7

**Published:** 2022-09-05

**Authors:** Chenchen Li, Haiyun Qin, Liuwang Zeng, Zhiping Hu, Chunli Chen

**Affiliations:** Department of Neurology, Second Xiangya Hospital, Central South University, Changsha, 410011 Hunan China

**Keywords:** Stem cells, ICH, Updated, meta-analysis, Animal models

## Abstract

**Background:**

Multiple studies have reported that stem cell therapy has beneficial effects in animal models of intracerebral hemorrhage (ICH). However, this finding remains inconclusive. This study was performed to systematically determine the effect size of stem cell therapy in ICH animal models by pooling and analyzing data from newly published studies.

**Methods:**

A literature search identified studies of stem cells in animal models of ICH. We searched mainstream databases from inception to November, 2021. And pooled effect size of stem cells was determined for diversified neurobehavioral scales and structural endpoints using random effects models.

**Results:**

The median quality score of 62 included studies was 5.32. Our results revealed an overall positive effect of stem cell therapy. More specifically, the SMD was − 2.27 for mNSS, − 2.14 for rotarod test, − 2.06 for MLPT, − 1.33 for cylinder test, − 1.95 for corner turn test, − 1.42 for tissue loss, and − 1.86 for brain water content. For mNSS, classifying comparisons by quality score showed significant differences in estimates of effect size (*p* = 0.013), and high-quality comparisons showed a better outcome (SMD = − 2.57) compared with low-quality comparisons (SMD = − 1.59). Besides, different delivery routes also showed a significant difference in the estimates of effect size for mNSS (*p* = 0.002), and the intraperitoneal route showed the best outcome (SMD = − 4.63). For tissue loss, the autologous blood-induced ICH model showed a better outcome (SMD = − 1.84) compared with the collagenase-induced ICH model (SMD = − 0.94, *p* = 0.035). Additionally, stem cell therapy initiated within 8 h post-ICH showed the greatest efficacy on tissue loss reduction, followed by initiated with 24 h post-ICH. Finally, stem cells with different sources and types showed similar beneficial effects for mNSS as well as tissue loss.

**Conclusions:**

Our results suggested that stem cell therapy had remarkable benefits on ICH animals on both the functional and structural outcomes in animal models of ICH, with very large effect size. These findings support the utility of further studies to translate stem cells in the treatment of ICH in humans. Moreover, the results should be interpreted in the light of the limitations in experimental design and the methodological quality of the studies included in the meta-analysis.

**Supplementary Information:**

The online version contains supplementary material available at 10.1186/s13287-022-03158-7.

## Introduction

Nontraumatic intracerebral hemorrhage (ICH) is highly associated with mortality and morbidity, with a substantially worse prognosis than ischemic stroke [1, 2]. The mortality rate of acute ICH is approximately 40% in the first three weeks, and those who survived often suffer from different degrees of neurological deficit [3]. To date, management of ICH is largely carried out via mechanical removal of the hematoma, decreasing intracranial pressure, controlling severe brain edema, and maintaining life function [4]. However, these approaches are not yet sufficiently effective to improve functional recovery after ICH [5, 6]. Therefore, new therapeutic strategies need to be explored under the guidance of evidence-based medicine.

There is now considerable preclinical literature on the possible benefits of stem cell transplantation following ICH [7, 8], yet there are some disputes over the safety and efficacy of stem cells [9]. Various types of stem cells, including neural stem cells (NSCs), immortalized pluripotent stem cells (IPSCs), mesenchymal stem cells (MSCs) and bone marrow-derived mononuclear cells (BM-MNCs), and bone marrow-derived endothelial progenitor cells (BM-EPCs), have attained tremendous attention as a promising approach in animal models of ICH [10–14]. They may assist stroke recovery through modulation of inflammation, angiogenesis, endogenous neurogenesis, and improved the blood–brain barrier integrity [15]. A prior meta-analysis by Hu and colleagues demonstrated the beneficial effects of stem cell therapy in animal models of ICH, but the database was only until April, 2015 [16]. Moreover, the results of the meta-analysis did not include “brain water content” as indicator of structural outcome. In recent years, an increasing number of studies have tried to explore the ideal subtype and dosage of stem cells, the appropriate time for injections, as well as the best administration route [17–19]. Thus, we aimed to perform an updated meta-analysis to provide the most comprehensive evidence relating to the therapeutic effects of stem cells on the functional and structural outcomes in animals exposed to ICH, and better foster the stem cell-based therapy that progressed into clinical translation.

## Methods

### Search strategy

Studies of stem cells in animal models of ICH were identified from the following mainstream databases (PubMed, EMBASE, and Web of Science) through November 18, 2021. Search terms were combined as follows: (progenitor cell OR stem cell OR bone marrow cell OR mesenchymal stromal cell OR mesenchymal cell OR hematopoietic cell) AND (intracerebral hemorrhage or hemorrhagic stroke or cerebral hemorrhage). Besides, the reference lists of eligible studies were also reviewed to identify other relevant articles.

### Inclusion and exclusion criteria

We included all controlled studies that compared stem cell therapy to vehicle or no-treatment in vivo models of ICH, in which the outcome was measured with neurobehavioral score or tissue loss or brain water content. To prevent bias, the inclusion criteria were prespecified as follows: (1) Experimental ICH was induced and the therapeutic effect of stem cells was assessed, no restriction on animal species, as well as gender, age, weight, and sample size; (2) Controlled studies with control group (receiving vehicle, saline, or no treatment) and experimental group (receiving xenogenic or allogeneic or syngeneic cell therapy), and there was no restriction on the dosage of stem cell, animal model of ICH, and time of initial treatment; (3) Studies that have neurobehavioral score or tissue loss or brain water content as outcome measurement. (4) There had to be full text available within a peer-reviewed journal, published in English. Articles that reported on the same sample were treated as a single study. Reviewers (Chenchen Li and Haiyun Qin) independently screened the abstracts according to the inclusion criteria, and disagreements were addressed in a discussion with a third reviewer (Chunli Chen). The meta-analysis excluded studies where we could not calculate the number of animals, the mean outcome, or the standard deviation (SD) in each group. We also excluded studies that used substantially manipulated stem cells, including cells differentiated into mature neural cells, co-treatment with another therapy, or transfected with overexpressed or underexpressed particular genes. However, stem cells that had been labeled or transfected with cellular markers intended for tracing and imaging were included.

### Data collection

The following items from the eligible studies were independently extracted by the two investigators (Chenchen Li and Haiyun Qin): general study information (first author, publication year); animal species, gender; anesthetics used; sample size; method of ICH induction; type and dose of stem cells; time of administration; route of delivery; follow-up time; functional outcome (neurobehavioral score measured on any scale), structural outcome (tissue loss or brain water content) and study quality index. When a publication reported more than one experiment or where an experiment contained more than one individual comparison, they were regarded as independent experiments, and data for every individual comparison from each experiment were extracted, respectively. If neurobehavioral tests were performed at different times, we only extracted data for the final time point reported. If the data from multiple brain slices were reported in brain water content, we only extracted the data of ipsilateral basal ganglia. If the SD was not directly reported, we calculated it by multiplying the reported standard error (SE) by the square root of the group size. Additionally, if data were only presented graphically, we measured values for the mean and SD from graphs using quantitative methods on highly magnified images (GetData Graph Digitizer, version 2.26). For each comparison, we extracted data regarding mean and SD from both the control and treatment groups to compare the efficacy of stem cell.

### Methodological quality of studies

The quality of each experiment was assessed according to the Collaborative Approach to Meta-Analysis and Review of Animal Data from Experimental Studies (CAMARADES) checklists, which consist of the following: (1) peer reviewed publication; (2) control of temperature; (3) random allocation to treatment or control; (4) blinded induction of hemorrhage; (5) blinded assessment of outcome; (6) use of anesthetic without marked intrinsic neuroprotective activity such as ketamine; (7) animal model with relevant comorbidities (aged, diabetic, or hypertensive); (8) sample size calculation; (9) compliance with animal welfare regulations; and (10) statement of potential conflict of interests. We defined studies that scored < 5 points were considered to be of low quality, and studies that scored ≥ 5 points were considered to be of high quality.

### Statistical analysis

We used Comprehensive Meta-analysis software (version 2; Biostat Inc) to perform all the statistical analyses. Mean, SD, and sample size were primarily used to generate effective sizes. For continuous endpoints, standardized mean difference (SMD) was calculated and presented with accompanying 95% confidence intervals. Random-effects models were chosen for the meta-analysis as we hypothesized that within-study and between-study moderators would result in differences of the true effect size [20]. It was a more conservative approach because the random-effects model yields a wider 95% confidence interval (CI) than the fixed effects model (which estimates study weight based on sample size). Effect size on the neurobehavioral score, tissue loss, and brain water content of stem cells were compared between the treatment and control groups. Besides, outcome values were multiplied by − 1 if a higher value indicates a more favorable outcome. Sensitivity analyses were performed by omitting one study at a time to evaluate whether the results were affected by a single study. The percentage of heterogeneity across the studies was estimated by I^2^ statistic. An I^2^ statistic of < 25% indicated low heterogeneity, 25% to 50% indicated moderate heterogeneity, and > 50% indicated high heterogeneity [21]. Publication bias was detected by funnel plotting. Asymmetry was assessed using an Egger’s test and the trim-and-fill method [22]. Finally, the prespecified subgroups were used to stratify the effect size: species, methods of ICH, delivery routes, cell sources (autologous, allogeneic, or xenogeneic); cell types (MSCs,NSCs, IPSCs or other stem cells); cell dosage (< 1 × 10^6^, [1–5] × 10^6^, > 5 × 10^6^); delivery time (24 h, [0–8 h], (1–7 days], or > 7 days); and quality index. A Q-test based on analyses of variance was used to assess the difference between subgroups of studies [23]. Statistical significance was set at *p* < 0.05 except where noted, and the 95% CIs of all results were calculated.

## Results

### Study selection

**Fig. 1:**
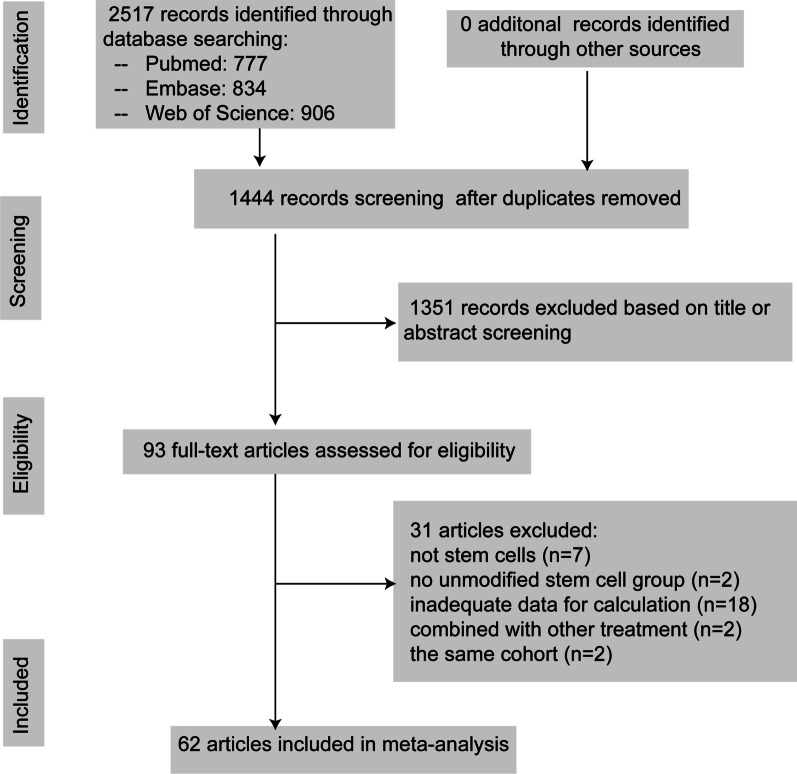
Flow diagram of literature search and study selection

The meta-analysis was conducted and reported in compliance with PRISMA ( preferred reporting items for systematic review and meta-analyses) guidelines [24]. The literature search identified 2517 potential studies at the primary retrieval: 777 records in PubMed, 834 in Embase and 906 in Web of Science. After review and exclusion, 93 full-text articles remained and were evaluated for inclusion eligibility. From these, 31 records were excluded due to the reasons given in Fig. 1. Finally, data from 62 studies published from 2003 to 2021 were included in the meta-analysis.

### Study characteristics

As depicted in Additional file 1: Table S1, the majority of the studies were carried out in rodents (rats and mice) except for one study that was conducted in monkeys [25]. The vast majority of studies (*n* = 44) used collagenase-induced model and autologous induced blood model (*n*  = 17) while one study applied hemoglobin [26]. The total dosage of stem cells ranged from 1.0 × 10^5^ to 1.0 × 10^9^, and the most frequent dose was 1 × 10^6^. Following the induction of ICH, stem cells were infused either immediately or over a period varying from 0.5 h to 2 months. The duration of follow-up ranged from 1 day to 6 months. The most common route used for infusing stem cells was the intracerebral route. Other routes used were the intravenous, intra-arterial, and intraperitoneal routes. Isoflurane, pentobarbital, ketamine, chloral hydrate, halothane, xylazine, and zoletil were the standard anesthetic agents used.

The vast majority of studies used MSCs and NSCs derived from mice, rats, or humans, and remaining studies used IPSCs, BM-MNCs, BM-EPCs, and umbilical cord blood-derived mononuclear cells (UCB-MNCs). Additionally, the type of MSCs include bone marrow mesenchymal stem cells (BMSCs), adipose-derived mesenchymal stromal cells (ADSCs), umbilical cord tissue-derived mesenchymal stem cells (UC-MSCs), umbilical cord blood-derived mesenchymal stem cells (UCB-MSCs), amniotic membrane mesenchymal stem cells (AMSCs), placenta-derived mesenchymal stem cells (PD-MSCs), and olfactory mucosa mesenchymal stem cells (OM-MSC). In addition, functional outcomes were assessed by modified neurological severity score (mNSS) or neurological severity score (NSS) in 49 comparisons (35 studies), rotarod test in 19 comparisons, corner turn test in 18 comparisons, modified limb placement test (MLPT) or LPT (forelimb placing test) in 18 comparisons, and cylinder test in 6 comparisons. Eighteen studies evaluated the structural outcomes, which was assessed by tissue loss or lesion volume in 30 comparisons and brain water content in 22 comparisons.

### Study quality

The quality score of the studies ranged from 2 to 8 (mean 5.32), among them 48 (77.42%) included studies were regarded as high methodological quality (≥ 5) studies. All studies have been published in peer-reviewed journals and stated compliance with animal welfare regulations. Fifty percentage studies reported describing control of temperature; 42 (67.74%) studies reported randomized allocation to treatment group; 44 (70.97%) studies reported blinded assessment of outcome. None of them used masked induction of hemorrhage. Three (4.84%) studies used animals with relevant comorbidities (e.g., hypertension). Four (6.45%) studies reported a sample size calculation. Forty-three (69.35%) studies avoided using anesthetics with known marked intrinsic neuroprotective properties such as ketamine. Thirty-eight (61.29%) studies stated possible conflicts of interest. The details of quality index are concluded in Additional file 2: Table S2.

### Global estimates of efficacy

**Fig. 2:**
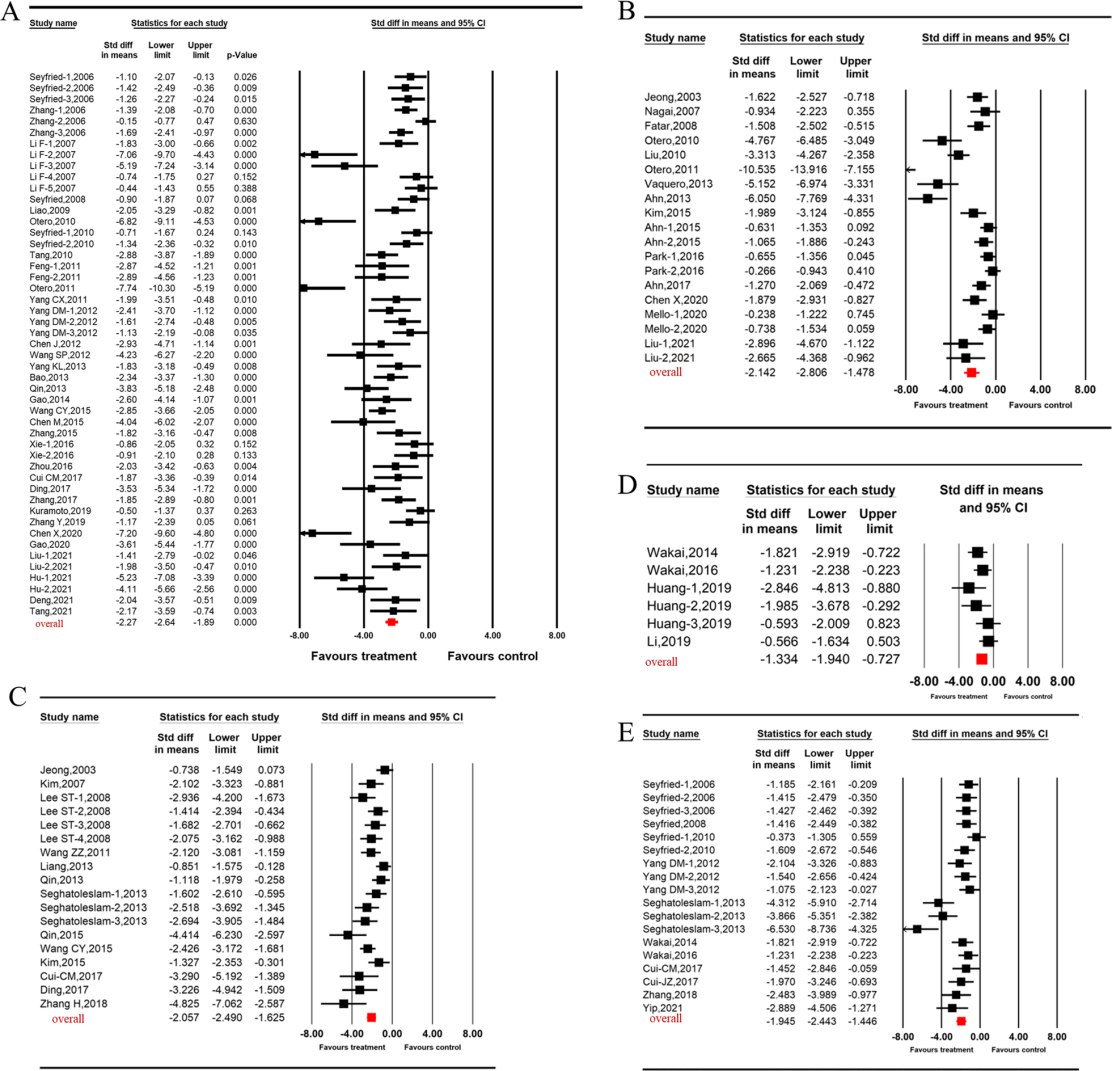
Forest plot shows mean effect size and 95% CI for (**A**) mNSS, (**B**) rotarod test, (**C**) MLPT, (**D**) cylinder test, (**E**) corner turn test between stem cell therapies treatment group and control group. SMD standardized mean difference; mNSS: modified neurological severity score; MLPT: modified limb placement test

**Fig. 3:**
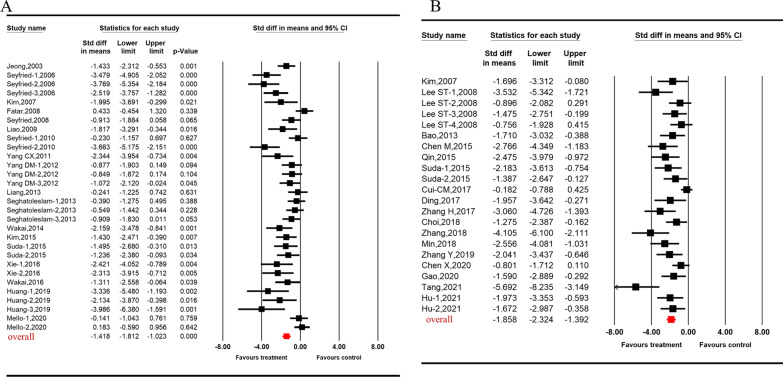
Forest plot shows mean effect size and 95% CI for (**A**) tissue loss, (**B**) brain water content between stem cell therapies treatment group and control group. SMD standardized mean difference

Overall, the pooled analysis showed the effect size of stem cells on functional and structural outcomes post-ICH were very large and of comparable magnitude. More specifically, the SMD was − 2.27 [95% CI (− 2.64, − 1.89), I^2^ = 78.31%, *p* < 0.001, Fig. 2A] for mNSS, − 2.14 [95% CI (− 2.81, − 1.48), I^2^ = 86.99%, *p* < 0.001,Fig. 2B] for rotarod test, − 2.06 [95% CI (− 2.49, − 1.63), I^2^ = 64.13%, *p* < 0.001, Fig. 2C] for MLPT, − 1.33 [95% CI (− 1.94, − 0.73), I^2^ = 24.90%, *p* < 0.001, Fig. 2D] for cylinder test, − 1.95 [95% CI (− 2.44, − 1.45), I^2^ = 67.76%, *p* < 0.001, Fig. 2E] for corner turn test, − 1.42 [95% CI (− 1.81, − 1.02), I^2^ = 70.76%, *p* < 0.001, Fig. 3A] for tissue loss, and − 1.86 [95% CI (− 2.32, − 1.39), I^2^ = 62.94%, *p* < 0.001, Fig. 3B] for brain water content. For the two outcomes with the largest amount of published data—mNSS and tissue loss —stratified analysis was used to explore potential contributions to heterogeneity of the parameters mentioned above.

### Sensitivity analysis

We conducted sensitivity analysis to evaluate the stability of the results by sequential omission of each study to see if heterogeneity between the studies existed. The pooled SMD of mNSS was not significantly affected by any study, nor was the tissue loss or brain water content (Additional file 3: Fig. S1; Additional file 4: Fig. S2; Additional file 5: Fig. S3).

### Publication bias

**Fig. 4:**
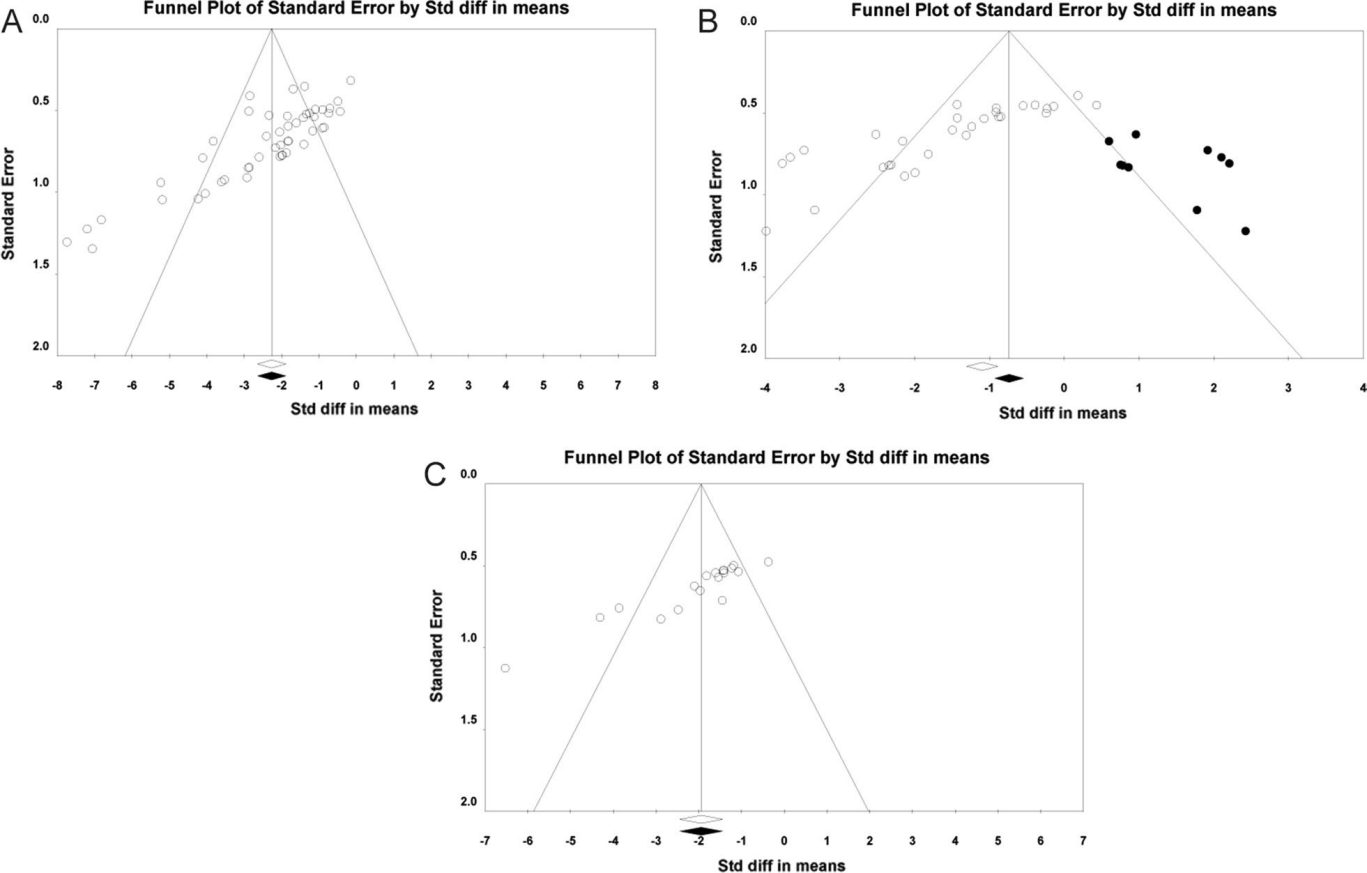
Funnel plots indicating possible publication bias for (**A**) mNSS (**B**) tissue loss, (**C**) brain water content. Open circles are included studies and black circles represent imputed studies from post hoc trim-and-fill analysis. mNSS: modified neurological severity score

Visual inspection of the funnel plots indicated potential publication bias for mNSS and brain water content (Fig. 4A&4C), which was confirmed by Egger’s regression test (*p* < 0.001). Then, we used the trim-and-fill method to estimate missing studies and recalculated the overall pooled effect estimates. Both of the imputed effect estimates of mNSS and brain water content were consistent with the previous one ( SMD = − 2.27, 95% CI − 2.64 to − 1.89, *p* < 0.001 for mNSS; SMD = − 1.86, 95% CI − 2.32 to − 1.39, *p* < 0.001 for brain water content, respectively), which implied no “missing” studies (Fig. 4A& C). On the other hand, the funnel plot also showed obvious asymmetry for the comparisons of tissue loss (Fig. 4B), and the results from Egger’s test confirmed significant publication bias (*p* < 0.001). After adopting trim-and-fill correction, the corrected estimates of tissue loss still remained statistically significant in favor of stem cell therapy, though reduced in magnitude of effect (SMD = − 0.83; 95% CI − 1.26 to − 0.40 for tissue loss, Fig. 4B).

### Stratified meta-analysis

For the two outcomes with the largest amount of published data—mNSS and tissue loss —stratified analysis was subsequently used to explore potential contributions to heterogeneity of the parameters mentioned above. The treatment effect was analyzed by pre-defined subgroups (species, methods of ICH, delivery routes, cell sources, cell types, cell doses, delivery time, and quality index). Figures of stratified analysis for mNSS and tissue loss were shown in Additional files 6 and 7. In general, the prominent efficacy of stem cell therapy was observed in most subgroups, but significance was not seen in a few individual subgroups (*p* ≥ 0.05).

For comparisons of mNSS, classifying studies between low and high-quality comparisons showed significant differences in estimates of effect size (*p* = 0.013), and high-quality comparisons showed a better outcome (SMD = − 2.57, 95% CI − 3.01 to − 2.13, *p* < 0.001) compared with low-quality comparisons (SMD = − 1.59, 95% CI − 2.22 to − 0.96, *p* < 0.001). Moreover, different delivery routes also showed significant differences in estimates of effect size (*p* = 0.002), and intraperitoneal route showed the best outcome (SMD = − 4.63, 95% CI − 6.46 to − 2.80, *p* < 0.001). Additionally, there was no significant difference among the comparisons with different species, different methods of ICH, different cell sources, different cell types, different delivery time, or different cell dose (Table 1).

**Table 1:** Stratified meta-analysis for mNSS

Categories	No. of comparisons	Pooled SMD (95% CI)	*p*-value	Heterogeneity test			Between groups *p*-value
				Q statistics	*I*^2^(%)	*p*_Q_-value	
Species							0.382
Rats	39	− 2.14 (− 2.56, − 1.72)	< 0.001	162.03	76.55	< 0.001	
Mice	8	− 2.81 (− 3.79, − 1.84)	< 0.001	53.85	87	< 0.001	
Monkey	2	− 2.88 (− 4.87, − 0.89)	0.005	0.001	0	0.982	
Quality							0.013*
High (score ≥ 5)	35	− 2.57 (− 3.01, − 2.13)	< 0.001	144.37	76.45	< 0.001	
Low (score < 5)	14	− 1.59 (− 2.22, − 0.96)	< 0.001	52.02	75.01	< 0.001	
Methods of ICH							0.196
Autologous blood	14	− 1.78 (− 2.47, − 1.09)	< 0.001	27.86	53.33	0.009	
Collagenase	34	− 2.46 (− 2.92, − 2.00)	< 0.001	189.08	82.55	0.00	
Hemoglobin	1	− 3.53 (− 6.44, − 0.62)	0.018	0.00	0.00	1.00	
Delivery routes							0.002*
Intra-arterial	7	− 1.91 (− 2.80, − 1.01)	< 0.001	37.65	84.06	< 0.001	
Intracerebral	22	− 2.72 (− 3.25, − 2.19)	< 0.001	74.08	71.65	< 0.001	
Intraperitoneal	2	− 4.63 (− 6.46, − 2.80)	< 0.001	0.84	0.00	0.361	
Intravenous	18	− 1.61 (− 2.15, − 1.07)	< 0.001	54.88	69.02	< 0.001	
Sources of stem cells							0.211
Allogeneic	24	− 2.50 (− 3.05, − 1.94)	< 0.001	141.69	83.77	< 0.001	
Syngeneic	3	− 3.16 (− 4.81, − 1.51)	< 0.001	19.32	89.65	< 0.001	
Xenogeneic	22	− 1.94 (− 2.50, − 1.38)	< 0.001	57.57	63.53	< 0.001	
Types of stem cells							0.622
BM-EPCs	1	− 1.85 (− 4.36, 0.67)	0.150	0.00	0.00	1.00	
IPSCs	1	− 3.83 (− 6.48, − 1.18)	0.005	0.00	0.00	1.00	
MSCs	40	− 2.20 (− 2.62, − 1.78)	< 0.001	168.85	76.90	< 0.001	
NSCs	7	− 2.52 (− 3.54, − 1.50)	< 0.001	42.61	85.92	< 0.001	
Time administration							0.667
[0–8 h]	13	− 2.48 (− 3.26, − 1.71)	< 0.001	33.78	64.48	0.001	
24 h	15	− 1.88 (− 2.57, − 1.20)	< 0.001	49.49	71.71	< 0.001	
(1 days–7 days]	15	− 2.33 (− 3.03, − 1.63)	< 0.001	80.46	82.60	< 0.001	
> 1 week	5	− 2.78 (− 4.05, − 1.51)	< 0.001	44.30	90.97	< 0.001	
NR	1	− 2.85 (− 5.33, − 0.38)	0.024	0.00	0.00	1.00	
Doses of stem cells							0.914
< 1 × 10^6^	16	− 2.29 (− 2.97, − 1.62)	< 0.001	39.57	62.10	0.001	
[1–5] × 10^6^	30	− 2.32 (− 2.81, − 1.83)	< 0.001	175.22	83.45	< 0.001	
> 5 × 10^6^	2	− 1.68 (− 3.52, 0.17)	0.075	1.04	3.99	0.307	
NR	1	− 1.87 (− 4.62, 0.87)	0.181	0.00	0.00	1.00	

*SMD* standardized mean difference, *CI* confidence interval, *ICH* intracerebral hemorrhage, *MSCs* mesenchymal stem cells, *NSCs* neural stem cells, *IPSCs* induced pluripotent stem cells, *BM-EPCs* bone marrow-derived endothelial progenitor cells, *NR* not reported

^*^Means *p* < 0.05

For comparisons of tissue loss, the results showed significant differences among different methods of the ICH model (*p* = 0.015, Table 2) as well as different delivery time (*p* = 0.035, Table 2). In detail, autologous blood-induced ICH model showed a better outcome (SMD = − 1.84, 95% CI − 2.36 to − 1.33, *p* < 0.001) compared with collagenase-induced ICH model (SMD = − 0.94, 95% CI − 1.46 to − 0.42, *p* < 0.001). Besides, stem cell therapy initiated within 8 h post-ICH showed the greatest effect size (SMD = − 2.72, 95% CI − 3.81 to − 1.62, *p* < 0.001), followed next by cell therapy initiated with 24 h (SMD = − 1.47, 95% CI − 2.29 to − 0.65, *p* < 0.001) and then therapy initiated more than 24 h (SMD = − 1.15, 95% CI − 1.60 to − 0.71, *p* < 0.001). In addition, there was no significant difference among the comparisons with different species, quality index, delivery routes, different cell sources, different cell types, or different cell dose (Table 2).

**Table 2:** Stratified meta-analysis for tissue loss

Categories	No. of studies	Pooled SMD (95% CI)	*p*-value	Heterogeneity test			Between groups *p*-value
				Q statistics	*I*^2^	*p*_Q_	
Species							0.687
Rats	28	− 1.40 (− 1.81, − 0.99)	< 0.001	96.48	72.01	< 0.001	
Mice	2	− 1.73 (− 3.28, − 0.18)	0.029	0.84	0.00	0.36	
Quality							0.128
High (score ≥ 5)	15	− 1.13 (− 1.67, − 0.58)	< 0.001	31.41	55.43	0.005	
Low (score < 5)	15	− 1.74 (− 2.31, − 1.17)	< 0.001	63.17	77.84	< 0.001	
Methods of ICH							0.015*
Autologous blood	16	− 1.84 (− 2.36, − 1.33)	< 0.001	46.41	67.68	< 0.001	
Collagenase	14	− 0.94 (− 1.46, − 0.42)	< 0.001	35.56	63.45	0.001	
Delivery routes							0.283
Intra-arterial	1	− 0.91 (− 2.93, 1.10)	0.374	0.00	0.00	1.00	
Intracerebral	10	− 1.91 (− 2.65, − 1.18)	< 0.001	16.86	46.63	0.051	
Intravenous	19	− 1.24 (− 1.72, − 0.76)	< 0.001	74.67	75.90	< 0.001	
Sources of Stem cells							0.872
Allogeneic	8	− 1.48 (− 2.25, − 0.70)	< 0.001	22.26	68.55	0.002	
Xenogeneic	22	− 1.40 (− 1.87, − 0.94)	< 0.001	76.28	72.47	< 0.001	
Types of stem cells							0.459
MNCs	5	− 0.89 (− 1.83, 0.04)	0.061	3.05	0.00	0.55	
MSCs	22	− 1.54 (− 2.02, − 1.06)	< 0.001	91.46	77.04	< 0.001	
NSCs	3	− 1.62 (− 2.86, − 0.37)	0.011	1.03	0.00	0.60	
Time administration							0.035*
[0–8 h]	5	− 2.72 (− 3.81, − 1.62)	< 0.001	2.17	0.00	0.70	
24 h	19	− 1.15 (− 1.60, − 0.71)	< 0.001	74.90	75.97	< 0.001	
(24 h–7 days]	6	− 1.47 (− 2.29, − 0.65)	< 0.001	4.11	0.00	0.53	
Doses of stem cells							0.578
< 1 × 10^6^	9	− 1.77 (− 2.54, − 1.00)	< 0.001	14.05	43.07	0.08	
[1–5] × 10^6^	18	− 1.30 (− 1.81, − 0.79)	< 0.001	73.57	76.89	< 0.001	
> 5 × 10^6^	3	− 1.26 (− 2.46, − 0.07)	0.038	6.68	70.06	0.035	

*SMD* standardized mean difference, *CI* confidence interval, *ICH* intracerebral hemorrhage, *MSCs* mesenchymal stem cells, *MNCs* mononuclear cells

^*^Means *p* < 0.05

## Discussion

### Summary of evidence

The current meta-analysis builds on prior meta-analyses of stem cell therapy, each of which had its own approach. Ma et al. reviewing studies up to 2014, and demonstrated the beneficial effects of stem cell therapy in animal models of ICH. However, they did not perform various stratified analyses except cell type [27]. Hu et al. reviewed studies up to 2015, provided key insights and focused on ICH, but they did not include the outcome “brain water content” as a structural indicator [16]. In recent years, more and more evidences which explored the ideal subtype and dose of stem cells, the appropriate time for injections, as well as the best administration route for animal models of ICH emerged. Thus, we aimed to provide the most updated and comprehensive evidence relating to the therapeutic effects of stem cells on the functional and structural outcomes in animals exposed to ICH in this meta-analysis. In general, our results suggested the following: (1) The results suggested that stem cell therapy showed remarkable benefits on ICH animals on diversified neurobehavioral scales and structural outcome indicators. More specifically, the SMD was − 2.27 for mNSS, − 2.14 for rotarod test, − 2.06 for MLPT, − 1.33 for cylinder test, − 1.95 for corner turn test, − 1.42 for tissue loss, and − 1.86 for brain water content. (2) For mNSS, classifying comparisons by quality score showed significant differences in the estimates of effect size (*p* = 0.013), and high-quality comparisons showed a better outcome (SMD =  − 2.57) compared with low-quality comparisons (SMD =  − 1.59). (3) For mNSS, different delivery routes also showed significant differences in the estimates of effect size for mNSS (*p* = 0.002), and intraperitoneal route showed the best outcome (SMD =  − 4.63). However, as the number of animals for the intraperitoneal route in the pooled analysis was small, this result needs to be proved by more studies. (4) For tissue loss, autologous blood-induced ICH model showed a better outcome (SMD =  − 1.84) compared with collagenase-induced ICH model (SMD = − 0.94, *p* = 0.035). In addition, stem cell therapy initiated within 8 h post-ICH showed the greatest efficacy on tissue loss reduction, followed by initiated with 24 h post-ICH, and then therapy initiated more than 24 h. (5) Stem cells with different sources and types showed similar beneficial effects for mNSS and tissue loss. (6) It is unclear from the included studies which is the ideal dosage of stem cell. Overall, the above various subgroup analyses can only generate hypotheses rather than confirming them.

### Possible mechanisms of stem cells on ICH

Numerous studies of stem cell therapy have been conducted to explore the exact mechanism in animal models of ICH. First, the anti-inflammatory role of transplanted stem cell was evidenced by their ability to decrease the number of microglial cells/macrophages and neutrophils in the perihematomal region and attenuate the expression of proinflammatory cytokines in the brain and/or plasma in animal models of ICH [11, 28, 29]. Besides the anti-inflammatory action, stem cells can increase the number of proliferating cells and decrease the number of apoptotic cells in the perihematomal region as well as upregulate the expression of antiapoptotic molecules [30, 31]. In addition, stem cells have been shown to release neurogenic cytokines or neurotrophic factors in a paracrine manner, which can stimulate endogenous neurogenesis and aid in reconstitution of neurovascular unit in perihematoma regions [32]. Moreover, stem cell therapy can increase the expression of tight junction proteins (zonula occludens-1 and claudin-5) and improve the blood–brain barrier integrity after ICH [12, 28]. Finally, the underlying mechanisms of stem cell therapy to accelerate neurological function recovery after ICH were also attributed to its effects of promoting angiogenesis [29, 33].

### Interpretation of subgroup analysis by study quality

The quality of preclinical stem cell studies was reviewed given the important bearing this has on translational potential. The median quality score value in the current study was 5.32, remarkably higher than the value of 4.45 reported by Hu et al. [16] using the same quality scale. The results in our meta-analysis showed higher study quality was associated with a larger effect size of mNSS related to stem cell therapy. This is consistent with the result of one recent meta-analysis for ischemia stroke [34]. The quality criteria that were not addressed in most of the included studies were concealment of hemorrhage allocation, sample size calculations, and testing of animals with relevant comorbidities. Previous studies showed that both a lack of sample size justification and allocation concealment in preclinical experimentation has had a detrimental influence on the estimation of true effect size [35, 36]. Therefore, we encourage future research to follow standardization and rigorous criteria of CAMARADES guidelines to minimize bias on methodology in the field.

### Interpretation of subgroup analysis by administration routes

In our present study, the administration routes included intracerebral injection, intra-arterial injection, intravenous injections, and intraperitoneal injection. Compared with intracerebral injection, the remaining injection routes are relatively less invasive and more convenient to manipulate. Temporarily disregarding the inconvenience of intracerebral injection, it does show superiorities over other routes because it can rapidly and directly target the lesion site while avoiding crossing the blood–brain barrier [37–39]. In our study, intraperitoneal injection seems to show the greatest efficacy for mNSS, followed by intracerebral injection, intra-arterial injection, and intravenous injections, but the small sample sizes and large confidence interval diminished the robustness of the subgroup data. So we need more research to clarify which administration route is better.

### Interpretation of subgroup analysis by methods of ICH induction

Two rodent models of ICH are most commonly used: injection of the enzyme collagenase and injection of autologous blood. In our study, autologous blood-induced ICH model showed a better outcome compared with collagenase-induced ICH model for tissue loss. Manaenko A. et al. [37] reported that hematoma size became larger in the stage of secondary brain injury and severe neurological dysfunction occurred in the collagenase-induced ICH model rather than autologous blood model. Besides, collagenase-induced ICH model produced greater edema, loss of cortical connections and secondary shrinkage of the striatum [38]. Finally, disruption of the blood–brain barrier caused by collagenase injection was significantly more serious in comparison with autologous blood model. Thus, the differences in pathophysiological mechanisms between these two models may help explain the better efficacy of stem cell therapy in the autologous blood model for tissue loss reduction.

### Interpretation of subgroup analysis by cell doses, source and time administration

Cell doses are typically the topic of concern when stem cell therapy is applied in clinical situations. However, due to the differences in the animal models used, type of transplanted cells, and cell isolation and purification techniques between different studies, the dose of cells administered varied greatly from one study to another. The included literature suggested that the stem cell dose administered in animal models of.

ICH was in the range from 1.0 × 10^5^ to 1.0 × 10^9^ and the most frequent dose was 1 × 10^6^. Consistent with the prior meta-analysis by Hu and colleagues [16], we did not find a significant difference in the effect size of mNSS among subgroups with different dose. In addition, no significant effect size of stem cell therapy on mNSS was observed in the higher dose subgroup (> 5 × 10^6^). A higher number of cells maybe a concern, since the risk of microembolism would increase, especially with large cells like MSCs [39, 40]. Besides, larger doses of stem cell might affect the organ perfusion and reduce cerebral blood flow [41, 42]. Last but not least, it should be interpreted with carefully because the higher dose subgroup only included a small number of comparisons.

The sources for stem cells may be autologous, syngeneic, or xenogeneic. Autologous stem cell transplantation guarantees the absence of immune rejection. However, depending on the circumstances, this might not be accessible to obtain autologous stem cells from some patients or the stem cells obtained from the elderly patients may have regenerative capabilities [43]. Obtaining and using syngeneic or xenogeneic stem cells is a way of resolving these issues. They are readily available, cost-effective, and high quality and hence serve as a promising alternative to stem cells from autologous sources. The previous meta-analysis by Fernandez et al. and Hu et.al found that both xenogeneic and syngeneic stem cells were equally efficacious in terms of functional and behavioral recovery for ischemia and hemorrhagic stroke, respectively [16, 44]. Consistent with the above-mentioned analysis, we demonstrated allogenic, syngeneic or xenogenic cells exhibited similarly beneficial effect size for mNSS and tissue loss. These results may suggest stem cells with different immunities could equally be used for ICH animal models.

It is also considerable to determine the role of stem cells in ICH models in the light of initial time administration. The majority of the included studies tended to initiate the cell therapy at 24 h post-ICH, followed by therapy initiation within 1–7 days post-ICH, initiation within 8 h and then therapy initiated more than 24 h. These favorable effects on the neurological recovery and tissue loss reduction was seen on all subgroups with different initial time, which suggested the therapeutic time window of stem cell transplantation might be wide enough for ICH. Besides, Vaquero et al. have shown that administration of BM-MSCs after two months post-ICH significantly improved the functional outcome of ICH rats [45, 46], which is promising for patients who are in chronic phases of ICH in clinical practice. In addition, our meta-analysis showed stem cell therapy initiated within 8 h post-ICH exhibited the greatest efficacy for tissue loss reduction, rather than initiated with 24 h post-ICH. In general, cytokines such as TNF-α activation can be detected early in a few hours after stroke [47, 48], even before significant neuronal death has occurred, and the spleen secretes large amounts of TNF-α into circulating blood at the hyper-acute stage [49]. Further, Growing pieces of evidence reported that the favorable effects of early stem cell administration can be largely attributed to their anti-inflammation effects in hyper-acute phase ICH [28, 48, 50, 51]. This may help explain the superior effects seen with stem cells in hyper-acute phase ICH rather than 24 h post-ICH.

### Strengths

This research took great efforts to ensure that the study can arrive at a relatively objective result and it had some strengths. First, our study provides an up-to-date meta-analysis of the effect size of stem cells in ICH animal models. Although previously published meta-analysis assessed the effectiveness of stem cell therapy in ICH animal models, our study tried to collect most recent reports in this field. Besides, we included studies of all kinds of stem cells on diversified neurobehavioral scales as well as structural outcome indicators for ICH animal models, therefore provided the most complete evidences of stem cells. Moreover, among the included 62 published studies, 48 (77.42%) included studies were regarded as high methodological quality (≥ 5) studies, and we conducted a thorough and careful literature search that obeyed publishing protocols to ensure a strict reviewing procedure. Third, numerous subgroup analyses based on the various animal models, species, sources of stem cell, routes of administration, dose of stem cells, and initial time of stem cell administration were conducted to explore potential contributions to heterogeneity. Finally, although funnel plots and Egger tests detected obvious publication bias, the modified effect sizes of both behavioral and structural outcomes were still significant after adopting trim-and-fill approach for correcting the bias.

### Limitations

On the other hand, the present systematic review and meta-analysis also had some limitations. First, although our search strategy was exhaustive, it is also possible that some published studies were missed. Second, the meta-analysis was limited to relatively small data sets. Although 2517 studies were identified by electronic searching, there were only 62 publications that met our criteria. Further studies with large sample sizes are warranted to provide sufficient evidence about the effects of stem cell therapy on ICH. Third, in our meta-analysis, most included studies did not examine the effects of stem cells in specific ICH populations with comorbidities such as aged or diabetes or hypertension, who may have different responses to stem cell therapy. Thus, there is significant work to be done when it comes to clinical translation.

### Clinical perspective

To date, a few clinical trials on stem cells have shown relatively modest benefits in ICH [52–57]. The majority of these trials have supported the safety, tolerability, and feasibility of stem cell transplantation in ICH. However, these clinical trials had obvious deficiencies, such as small sample size, lack of control group, as well as only including patients with cerebral hemorrhage sequela. Moreover, the optimal choice of the source, dose, and transplantation route of stem cells is inconclusive. As many open issues are still unsolved, larger and longer-term trials are warranted to determine the more favorable parameter of stem cell transplantation and to further evaluate the potential of stem cell therapy in ICH.

## Conclusions

Our current meta-analysis demonstrates that stem cell treatment significantly improves both functional and structural outcomes in animal models of ICH. A number of factors support translation to humans, including robustness of preclinical findings across variables such as species studied, sources of stem cells, time administration after ICH, and route of administration. As there is still a considerable degree of unexplained heterogeneity after using restrictive inclusion criteria for study selection, standardization of experiment design and measurement of preclinical experimentation for various functional as well as structural and outcomes of ICH are warranted in the future.

## Supplementary Information

**Additional file 1. Table S1**. Characteristics of 62 included studies.

**Additional file 2. Table S2**. Methodological quality of 62 studies included in the meta-analysis.

**Additional file 3**. Funnel plot of sensitivity analysis for mNSS.

**Additional file 4**. Funnel plot of sensitivity analysis for tissue loss.

**Additional file 5**. Funnel plot of sensitivity analysis for brain water content.

**Additional file 6**. Subgroup analysis for mNSS.

**Additional file 7**. Subgroup analysis for tissue loss.

## Data Availability

The original contributions presented in the study are included in the article and supplementary materials. Further inquiries can be directed to the corresponding author.

## Abbreviations

ICH: Intracerebral hemorrhage
SD: Standard deviation
SE: Standard error
SMD: Standardized mean difference
CI: Confidence interval
PRISMA: Preferred reporting items for systematic review and meta-analyses
CAMARADES: Collaborative Approach to Meta-Analysis and Review of Animal Data from Experimental Studies
mNSS: Modified neurological severity score
MLPT: Modified limb placement test
MHNs: Mesenchymal stem cells
NSCs: Neural stem cells
BMSCs: Bone marrow mesenchymal stem cells
iPSCs: Induced pluripotent stem cells
ADSCs: Adipose-derived mesenchymal stromal cells
UC-MSC: Umbilical cord tissue-derived mesenchymal stem cells
UCB-MSCs: Umbilical cord blood-derived mesenchymal stem cells
UCB-MNCs: Umbilical cord blood-derived mononuclear cells
BM-MNCs: Bone marrow-derived mononuclear cells
AHNMSCs: Amniotic membrane mesenchymal stem cells
BM-EPCs: Bone marrow-derived endothelial progenitor cells
D-MSCs: Placenta-derived mesenchymal stem cells
OM-MSC: Olfactory mucosa mesenchymal stem cells
