# Supplementary material for: Efficacy of stem cell therapy in animal models of intracerebral hemorrhage: an updated meta-analysis
Source: Stem Cell Res Ther. 2022 Sep 5;13:452. doi: 10.1186/s13287-022-03158-7 (PMC9446670; doi:10.1186/s13287-022-03158-7)
Supplement: Supplementary file 2 — Additional file 2. Table S2. Methodological quality of 62 studies included in the meta-analysis. [file 13287_2022_3158_MOESM2_ESM.doc]

Table S2. Methodological quality of 62 studies included in the meta-analysis

| Author year | peer reviewed publication | control of temperature | random allocation to treatment or control | blinded induction of haemorrhage  allocation concealment | blinded assessment  of outcome | use of anaesthetic without marked intrinsic neuroprotective activity | animal model (aged, diabetic or hypertensive) | sample size calculation | compliance with animal welfare regulations | conflict of interests | total score |
| --- | --- | --- | --- | --- | --- | --- | --- | --- | --- | --- | --- |
| Jeong et al. 2003[1] | √ | √ | √ |  | √ |  |  |  | √ |  | 5 |
| Seyfried et al.2006[2] | √ |  |  |  |  |  |  |  | √ |  | 2 |
| Zhang et al.2006[3] | √ |  | √ |  |  |  |  |  | √ |  | 3 |
| Nagai et al.2007[4] | √ | √ |  |  |  |  |  |  | √ | √ | 4 |
| Kim et al. 2007[5] | √ |  |  |  | √ |  |  |  | √ |  | 3 |
| Li F et al. 2007[6] | √ |  | √ |  | √ | √ |  |  | √ |  | 5 |
| Lee ST et al. 2008[7] | √ |  |  |  |  |  |  |  | √ | √ | 3 |
| Fatar et al. 2008[8] | √ |  | √ |  |  | √ |  |  | √ |  | 4 |
| Seyfried et al.2008[9] | √ |  |  |  |  | √ |  |  | √ |  | 3 |
| Liao.2009[10] | √ | √ |  |  | √ | √ |  |  | √ |  | 5 |
| Otero et al.2010[11] | √ |  | √ |  |  | √ |  |  | √ |  | 4 |
| Liu et al.2010[12] | √ | √ | √ |  |  |  |  |  | √ | √ | 5 |
| Seyfried et al.2010[13] | √ | √ | √ |  |  |  |  |  | √ |  | 4 |
| Tang et al.2010[14] | √ |  | √ |  |  | √ |  |  | √ |  | 4 |
| Feng et al.2011[15] | √ | √ | √ |  | √ |  |  |  | √ |  | 5 |
| Otero et al.2011[16] | √ |  | √ |  |  | √ |  |  | √ | √ | 5 |
| Yang CX et al.2011[17] | √ |  |  |  | √ | √ |  |  | √ | √ | 5 |
| Wang ZZ et al.2011[18] | √ |  | √ |  | √ | √ |  |  | √ | √ | 6 |
| Yang DM et al.2012[19] | √ |  | √ |  | √ |  |  |  | √ |  | 4 |
| Chen et al.2012[20] | √ | √ | √ |  | √ | √ |  |  | √ | √ | 7 |
| Wang SP et al.2012[21] | √ |  | √ |  | √ | √ |  |  | √ |  | 5 |
| Yang KL et al.2012[22] | √ | √ | √ |  | √ | √ |  |  | √ |  | 6 |
| Bao et al.2013[23] | √ | √ | √ |  | √ | √ |  |  | √ |  | 6 |
| Liang et al.2013[24] | √ | √ |  |  | √ | √ |  |  | √ |  | 5 |
| Qin et al.2013[25] | √ |  | √ |  | √ |  |  |  | √ | √ | 5 |
| Vaquero et al.2013[26] | √ |  | √ |  |  | √ |  |  | √ | √ | 5 |
| Ahn et al.2013[27] | √ |  |  |  | √ | √ |  |  | √ | √ | 5 |
| Seghatoleslam et al.2013[28] | √ | √ | √ |  | √ |  |  |  | √ |  | 5 |
| Gao et al.2014[29] | √ | √ |  |  | √ | √ |  |  | √ | √ | 6 |
| Wakai et al.2014[30] | √ | √ |  |  | √ | √ |  |  | √ | √ | 6 |
| Wang CY et al.2015[31] | √ |  |  |  | √ | √ | √ |  | √ | √ | 6 |
| Kim et al.2015[32] | √ |  |  |  |  | √ |  |  | √ |  | 3 |
| Chen M et al.2015[33] | √ |  | √ |  | √ | √ |  |  | √ | √ | 6 |
| Zhang et al.2015[34] | √ |  | √ |  | √ | √ |  |  | √ | √ | 6 |
| Ahn et al.2015[35] | √ | √ |  |  | √ |  |  |  | √ | √ | 5 |
| Qin et al.2015[36] | √ | √ | √ |  | √ |  |  |  | √ | √ | 6 |
| Suda et al.2015[37] | √ | √ |  |  | √ | √ | √ |  | √ | √ | 7 |
| Xie et al.2016[38] | √ | √ |  |  | √ | √ |  |  | √ |  | 5 |
| Wakai et al.2016[39] | √ |  |  | √ | √ | √ |  |  | √ | √ | 6 |
| Park et al.2016[40] | √ |  | √ |  | √ | √ |  | √ | √ | √ | 7 |
| Zhou et al.2016[41] | √ |  | √ |  | √ | √ |  |  | √ |  | 5 |
| Cui CM et al.2017[42] | √ | √ | √ |  | √ | √ |  |  | √ |  | 6 |
| Ding et al.2017[43] | √ |  | √ |  | √ | √ | √ |  | √ |  | 6 |
| Cui JZ et al.2017[44] | √ |  | √ |  | √ | √ |  |  | √ | √ | 6 |
| Ahn et al.2017[45] | √ | √ | √ |  | √ | √ |  | √ | √ | √ | 8 |
| Zhang et al.2017[46] | √ |  | √ |  |  | √ |  |  | √ | √ | 5 |
| Choi et al.2018[47] | √ | √ | √ |  | √ | √ |  | √ | √ | √ | 8 |
| Zhang H et al.2018[48] | √ |  | √ |  | √ | √ |  | √ | √ | √ | 7 |
| Min et al.2018[49] | √ | √ |  |  |  |  |  |  | √ | √ | 4 |
| Li et al.2019[50] | √ |  | √ |  | √ | √ |  |  | √ | √ | 6 |
| Huang et al.2019[51] | √ |  | √ |  |  |  |  |  | √ | √ | 4 |
| Zhang Y et al.2019[52] | √ | √ | √ |  | √ | √ |  |  | √ | √ | 7 |
| Kuramoto et al.2019[53] | √ |  | √ |  | √ | √ |  |  | √ | √ | 6 |
| Gao et al.2020[54] | √ | √ | √ |  | √ | √ |  |  | √ | √ | 7 |
| Mello et al  .2020[55] | √ | √ | √ |  | √ |  |  |  | √ | √ | 6 |
| Chen X et al. 2020[56] | √ | √ | √ |  | √ |  |  |  | √ | √ | 6 |
| Hu et al.2021[57] | √ | √ | √ |  | √ | √ |  |  | √ |  | 6 |
| Liu et al.2021-1[58] | √ | √ | √ |  | √ | √ |  |  | √ | √ | 7 |
| Liu et al.2021-2[59] | √ | √ |  |  | √ | √ |  |  | √ | √ | 6 |
| Deng et al. 2021[60] | √ | √ | √ |  | √ | √ |  |  | √ | √ | 7 |
| Tang et al. 2021[61] | √ | √ | √ |  |  |  |  |  | √ | √ | 5 |
| Yip et al. 2021[62] | √ | √ |  |  |  | √ |  |  | √ | √ | 5 |

**References:**

1. Jeong SW, Chu K, Jung KH, Kim SU, Kim M, Roh JK. Human neural stem cell transplantation promotes functional recovery in rats with experimental intracerebral hemorrhage. Stroke. 2003; 34: 2258-63.

2. Seyfried D, Ding J, Han Y, Li Y, Chen J, Chopp M. Effects of intravenous administration of human bone marrow stromal cells after intracerebral hemorrhage in rats. J Neurosurg. 2006; 104: 313-8.

3. Zhang H, Huang Z, Xu Y, Zhang S. Differentiation and neurological benefit of the mesenchymal stem cells transplanted into the rat brain following intracerebral hemorrhage. Neurol Res. 2006; 28: 104-12.

4. Nagai A, Kim WK, Lee HJ, Jeong HS, Kim KS, Hong SH, Park IH, Kim SU. Multilineage potential of stable human mesenchymal stem cell line derived from fetal marrow. PLoS One. 2007; 2: e1272.

5. Kim JM, Lee ST, Chu K, Jung KH, Song EC, Kim SJ, Sinn DI, Kim JH, Park DK, Kang KM, Hyung Hong N, Park HK, Won CH, et al. Systemic transplantation of human adipose stem cells attenuated cerebral inflammation and degeneration in a hemorrhagic stroke model. Brain Res. 2007; 1183: 43-50.

6. Li F, Liu Y, Zhu S, Wang X, Yang H, Liu C, Zhang Y, Zhang Z. Therapeutic time window and effect of intracarotid neural stem cells transplantation for intracerebral hemorrhage. Neuroreport. 2007; 18: 1019-23.

7. Lee ST, Chu K, Jung KH, Kim SJ, Kim DH, Kang KM, Hong NH, Kim JH, Ban JJ, Park HK, Kim SU, Park CG, Lee SK, et al. Anti-inflammatory mechanism of intravascular neural stem cell transplantation in haemorrhagic stroke. Brain. 2008; 131: 616-29.

8. Fatar M, Stroick M, Griebe M, Marwedel I, Kern S, Bieback K, Giesel FL, Zechmann C, Kreisel S, Vollmar F, Alonso A, Back W, Meairs S, et al. Lipoaspirate-derived adult mesenchymal stem cells improve functional outcome during intracerebral hemorrhage by proliferation of endogenous progenitor cells stem cells in intracerebral hemorrhages. Neurosci Lett. 2008; 443: 174-8.

9. Seyfried DM, Han Y, Yang D, Ding J, Savant-Bhonsale S, Shukairy MS, Chopp M. Mannitol enhances delivery of marrow stromal cells to the brain after experimental intracerebral hemorrhage. Brain Res. 2008; 1224: 12-9.

10. Liao W, Zhong J, Yu J, Xie J, Liu Y, Du L, Yang S, Liu P, Xu J, Wang J, Han Z, Han ZC. Therapeutic benefit of human umbilical cord derived mesenchymal stromal cells in intracerebral hemorrhage rat: implications of anti-inflammation and angiogenesis. Cell Physiol Biochem. 2009; 24: 307-16.

11. Otero L, Bonilla C, Aguayo C, Zurita M, Vaquero J. Intralesional administration of allogeneic bone marrow stromal cells reduces functional deficits after intracerebral hemorrhage. Histol Histopathol. 2010; 25: 453-61.

12. Liu AM, Lu G, Tsang KS, Li G, Wu Y, Huang ZS, Ng HK, Kung HF, Poon WS. Umbilical cord-derived mesenchymal stem cells with forced expression of hepatocyte growth factor enhance remyelination and functional recovery in a rat intracerebral hemorrhage model. Neurosurgery. 2010; 67: 357-65; discussion 65-6.

13. Seyfried DM, Han Y, Yang D, Ding J, Shen LH, Savant-Bhonsale S, Chopp M. Localization of bone marrow stromal cells to the injury site after intracerebral hemorrhage in rats. J Neurosurg. 2010; 112: 329-35.

14. Tang ZP, Xie XW, Shi YH, Liu N, Zhu SQ, Li ZW, Chen Y. Combined transplantation of neural stem cells and olfactory ensheathing cells improves the motor function of rats with intracerebral hemorrhage. Biomed Environ Sci. 2010; 23: 62-7.

15. Feng M, Zhu H, Zhu Z, Wei J, Lu S, Li Q, Zhang N, Li G, Li F, Ma W, An Y, Zhao RC, Qin C, et al. Serial 18F-FDG PET demonstrates benefit of human mesenchymal stem cells in treatment of intracerebral hematoma: a translational study in a primate model. J Nucl Med. 2011; 52: 90-7.

16. Otero L, Zurita M, Bonilla C, Aguayo C, Vela A, Rico MA, Vaquero J. Late transplantation of allogeneic bone marrow stromal cells improves neurologic deficits subsequent to intracerebral hemorrhage. Cytotherapy. 2011; 13: 562-71.

17. Yang C, Zhou L, Gao X, Chen B, Tu J, Sun H, Liu X, He J, Liu J, Yuan Q. Neuroprotective effects of bone marrow stem cells overexpressing glial cell line-derived neurotrophic factor on rats with intracerebral hemorrhage and neurons exposed to hypoxia/reoxygenation. Neurosurgery. 2011; 68: 691-704.

18. Wang Z, Cui C, Li Q, Zhou S, Fu J, Wang X, Zhuge Q. Intracerebral transplantation of foetal neural stem cells improves brain dysfunction induced by intracerebral haemorrhage stroke in mice. J Cell Mol Med. 2011; 15: 2624-33.

19. Yang D, Han Y, Zhang J, Seyda A, Chopp M, Seyfried DM. Therapeutic effect of human umbilical tissue-derived cell treatment in rats with experimental intracerebral hemorrhage. Brain Res. 2012; 1444: 1-10.

20. Chen J, Tang YX, Liu YM, Chen J, Hu XQ, Liu N, Wang SX, Zhang Y, Zeng WG, Ni HJ, Zhao B, Chen YF, Tang ZP. Transplantation of adipose-derived stem cells is associated with neural differentiation and functional improvement in a rat model of intracerebral hemorrhage. CNS Neurosci Ther. 2012; 18: 847-54.

21. Wang SP, Wang ZH, Peng DY, Li SM, Wang H, Wang XH. Therapeutic effect of mesenchymal stem cells in rats with intracerebral hemorrhage: reduced apoptosis and enhanced neuroprotection. Mol Med Rep. 2012; 6: 848-54.

22. Yang KL, Lee JT, Pang CY, Lee TY, Chen SP, Liew HK, Chen SY, Chen TY, Lin PY. Human adipose-derived stem cells for the treatment of intracerebral hemorrhage in rats via femoral intravenous injection. Cell Mol Biol Lett. 2012; 17: 376-92.

23. Bao XJ, Liu FY, Lu S, Han Q, Feng M, Wei JJ, Li GL, Zhao RC, Wang RZ. Transplantation of Flk-1+ human bone marrow-derived mesenchymal stem cells promotes behavioral recovery and anti-inflammatory and angiogenesis effects in an intracerebral hemorrhage rat model. Int J Mol Med. 2013; 31: 1087-96.

24. Liang H, Yin Y, Lin T, Guan D, Ma B, Li C, Wang Y, Zhang X. Transplantation of bone marrow stromal cells enhances nerve regeneration of the corticospinal tract and improves recovery of neurological functions in a collagenase-induced rat model of intracerebral hemorrhage. Mol Cells. 2013; 36: 17-24.

25. Qin J, Gong G, Sun S, Qi J, Zhang H, Wang Y, Wang N, Wang QM, Ji Y, Gao Y, Shi C, Yang B, Zhang Y, et al. Functional recovery after transplantation of induced pluripotent stem cells in a rat hemorrhagic stroke model. Neurosci Lett. 2013; 554: 70-5.

26. Vaquero J, Otero L, Bonilla C, Aguayo C, Rico MA, Rodriguez A, Zurita M. Cell therapy with bone marrow stromal cells after intracerebral hemorrhage: impact of platelet-rich plasma scaffolds. Cytotherapy. 2013; 15: 33-43.

27. Ahn SY, Chang YS, Sung DK, Sung SI, Yoo HS, Lee JH, Oh WI, Park WS. Mesenchymal stem cells prevent hydrocephalus after severe intraventricular hemorrhage. Stroke. 2013; 44: 497-504.

28. Seghatoleslam M, Jalali M, Nikravesh MR, Hamidi Alamdari D, Hosseini M, Fazel A. Intravenous administration of human umbilical cord blood-mononuclear cells dose-dependently relieve neurologic deficits in rat intracerebral hemorrhage model. Ann Anat. 2013; 195: 39-49.

29. Gao L, Lu Q, Huang LJ, Ruan LH, Yang JJ, Huang WL, ZhuGe WS, Zhang YL, Fu B, Jin KL, ZhuGe QC. Transplanted neural stem cells modulate regulatory T, γδ T cells and corresponding cytokines after intracerebral hemorrhage in rats. Int J Mol Sci. 2014; 15: 4431-41.

30. Wakai T, Sakata H, Narasimhan P, Yoshioka H, Kinouchi H, Chan PH. Transplantation of neural stem cells that overexpress SOD1 enhances amelioration of intracerebral hemorrhage in mice. J Cereb Blood Flow Metab. 2014; 34: 441-9.

31. Wang C, Fei Y, Xu C, Zhao Y, Pan Y. Bone marrow mesenchymal stem cells ameliorate neurological deficits and blood-brain barrier dysfunction after intracerebral hemorrhage in spontaneously hypertensive rats. Int J Clin Exp Pathol. 2015; 8: 4715-24.

32. Kim K, Park HW, Moon HE, Kim JW, Bae S, Chang JW, Oh W, Yang YS, Paek SH. The Effect of Human Umbilical Cord Blood-Derived Mesenchymal Stem Cells in a Collagenase-Induced Intracerebral Hemorrhage Rat Model. Exp Neurobiol. 2015; 24: 146-55.

33. Chen M, Li X, Zhang X, He X, Lai L, Liu Y, Zhu G, Li W, Li H, Fang Q, Wang Z, Duan C. The inhibitory effect of mesenchymal stem cell on blood-brain barrier disruption following intracerebral hemorrhage in rats: contribution of TSG-6. J Neuroinflammation. 2015; 12: 61.

34. Zhang Q, Shang X, Hao M, Zheng M, Li Y, Liang Z, Cui Y, Liu Z. Effects of human umbilical cord mesenchymal stem cell transplantation combined with minimally invasive hematoma aspiration on intracerebral hemorrhage in rats. Am J Transl Res. 2015; 7: 2176-86.

35. Ahn SY, Chang YS, Sung DK, Sung SI, Yoo HS, Im GH, Choi SJ, Park WS. Optimal Route for Mesenchymal Stem Cells Transplantation after Severe Intraventricular Hemorrhage in Newborn Rats. PLoS One. 2015; 10: e0132919.

36. Qin J, Ma X, Qi H, Song B, Wang Y, Wen X, Wang QM, Sun S, Li Y, Zhang R, Liu X, Hou H, Gong G, et al. Transplantation of Induced Pluripotent Stem Cells Alleviates Cerebral Inflammation and Neural Damage in Hemorrhagic Stroke. PLoS One. 2015; 10: e0129881.

37. Suda S, Yang B, Schaar K, Xi X, Pido J, Parsha K, Aronowski J, Savitz SI. Autologous Bone Marrow Mononuclear Cells Exert Broad Effects on Short- and Long-Term Biological and Functional Outcomes in Rodents with Intracerebral Hemorrhage. Stem Cells Dev. 2015; 24: 2756-66.

38. Xie J, Wang B, Wang L, Dong F, Bai G, Liu Y. Intracerebral and Intravenous Transplantation Represents a Favorable Approach for Application of Human Umbilical Cord Mesenchymal Stromal Cells in Intracerebral Hemorrhage Rats. Med Sci Monit. 2016; 22: 3552-61.

39. Wakai T, Narasimhan P, Sakata H, Wang E, Yoshioka H, Kinouchi H, Chan PH. Hypoxic preconditioning enhances neural stem cell transplantation therapy after intracerebral hemorrhage in mice. J Cereb Blood Flow Metab. 2016; 36: 2134-45.

40. Park WS, Sung SI, Ahn SY, Sung DK, Im GH, Yoo HS, Choi SJ, Chang YS. Optimal Timing of Mesenchymal Stem Cell Therapy for Neonatal Intraventricular Hemorrhage. Cell Transplant. 2016; 25: 1131-44.

41. Zhou H, Zhang H, Yan Z, Xu R. Transplantation of human amniotic mesenchymal stem cells promotes neurological recovery in an intracerebral hemorrhage rat model. Biochem Biophys Res Commun. 2016; 475: 202-8.

42. Cui C, Cui Y, Gao J, Li R, Jiang X, Tian Y, Wang K, Cui J. Intraparenchymal treatment with bone marrow mesenchymal stem cell-conditioned medium exerts neuroprotection following intracerebral hemorrhage. Mol Med Rep. 2017; 15: 2374-82.

43. Ding R, Lin C, Wei S, Zhang N, Tang L, Lin Y, Chen Z, Xie T, Chen X, Feng Y, Wu L. Therapeutic Benefits of Mesenchymal Stromal Cells in a Rat Model of Hemoglobin-Induced Hypertensive Intracerebral Hemorrhage. Mol Cells. 2017; 40: 133-42.

44. Cui J, Cui C, Cui Y, Li R, Sheng H, Jiang X, Tian Y, Wang K, Gao J. Bone Marrow Mesenchymal Stem Cell Transplantation Increases GAP-43 Expression via ERK1/2 and PI3K/Akt Pathways in Intracerebral Hemorrhage. Cell Physiol Biochem. 2017; 42: 137-44.

45. Ahn SY, Chang YS, Sung DK, Sung SI, Ahn JY, Park WS. Pivotal Role of Brain-Derived Neurotrophic Factor Secreted by Mesenchymal Stem Cells in Severe Intraventricular Hemorrhage in Newborn Rats. Cell Transplant. 2017; 26: 145-56.

46. Zhang R, Yang J, Yuan J, Song B, Wang Y, Xu Y. The Therapeutic Value of Bone Marrow-Derived Endothelial Progenitor Cell Transplantation after Intracerebral Hemorrhage in Rats. Front Neurol. 2017; 8: 174.

47. Choi BY, Kim OJ, Min SH, Jeong JH, Suh SW, Chung TN. Human Placenta-Derived Mesenchymal Stem Cells Reduce Mortality and Hematoma Size in a Rat Intracerebral Hemorrhage Model in an Acute Phase. Stem Cells Int. 2018; 2018: 1658195.

48. Zhang H, Wang Y, Lv Q, Gao J, Hu L, He Z. MicroRNA-21 Overexpression Promotes the Neuroprotective Efficacy of Mesenchymal Stem Cells for Treatment of Intracerebral Hemorrhage. Front Neurol. 2018; 9: 931.

49. Min S, Kim OJ, Bae J, Chung TN. Effect of Pretreatment with the NADPH Oxidase Inhibitor Apocynin on the Therapeutic Efficacy of Human Placenta-Derived Mesenchymal Stem Cells in Intracerebral Hemorrhage. Int J Mol Sci. 2018; 19.

50. Li G, Yu H, Liu N, Zhang P, Tang Y, Hu Y, Zhang Y, Pan C, Deng H, Wang J, Li Q, Tang Z. Overexpression of CX3CR1 in Adipose-Derived Stem Cells Promotes Cell Migration and Functional Recovery After Experimental Intracerebral Hemorrhage. Front Neurosci. 2019; 13: 462.

51. Huang P, Freeman WD, Edenfield BH, Brott TG, Meschia JF, Zubair AC. Safety and Efficacy of Intraventricular Delivery of Bone Marrow-Derived Mesenchymal Stem Cells in Hemorrhagic Stroke Model. Sci Rep. 2019; 9: 5674.

52. Zhang Y, Deng H, Hu Y, Pan C, Wu G, Li Q, Tang Z. Adipose-derived mesenchymal stem cells stereotactic transplantation alleviate brain edema from intracerebral hemorrhage. J Cell Biochem. 2019; 120: 14372-82.

53. Kuramoto Y, Takagi T, Tatebayashi K, Beppu M, Doe N, Fujita M, Yoshimura S. Intravenous administration of human adipose-derived stem cells ameliorates motor and cognitive function for intracerebral hemorrhage mouse model. Brain Res. 2019; 1711: 58-67.

54. Gao L, Li PP, Shao TY, Mao X, Qi H, Wu BS, Shan M, Ye L, Cheng HW. Neurotoxic role of interleukin-17 in neural stem cell differentiation after intracerebral hemorrhage. Neural Regen Res. 2020; 15: 1350-9.

55. Mello TG, Rosado-de-Castro PH, Campos RMP, Vasques JF, Rangel-Junior WS, Mattos R, Puig-Pijuan T, Foerster BU, Gutfilen B, Souza SAL, Boltze J, Paiva FF, Mendez-Otero R, et al. Intravenous Human Umbilical Cord-Derived Mesenchymal Stromal Cell Administration in Models of Moderate and Severe Intracerebral Hemorrhage. Stem Cells Dev. 2020; 29: 586-98.

56. Chen X, Liang H, Xi Z, Yang Y, Shan H, Wang B, Zhong Z, Xu C, Yang GY, Sun Q, Sun Y, Bian L. BM-MSC Transplantation Alleviates Intracerebral Hemorrhage-Induced Brain Injury, Promotes Astrocytes Vimentin Expression, and Enhances Astrocytes Antioxidation via the Cx43/Nrf2/HO-1 Axis. Front Cell Dev Biol. 2020; 8: 302.

57. Hu J, Chang Y, Peng C, Huang S, Li G, Li H. Umbilical Cord Mesenchymal Stem Cells Derived Neurospheres Promote Long-term functional recovery But Aggravate Acute Phase Inflammation in Experimental Stroke. Neuroscience. 2022; 480: 217-28.

58. Liu J, He J, Huang Y, Ge L, Xiao H, Zeng L, Jiang Z, Lu M, Hu Z. Hypoxia-preconditioned mesenchymal stem cells attenuate microglial pyroptosis after intracerebral hemorrhage. Ann Transl Med. 2021; 9: 1362.

59. Liu J, He J, Ge L, Xiao H, Huang Y, Zeng L, Jiang Z, Lu M, Hu Z. Hypoxic preconditioning rejuvenates mesenchymal stem cells and enhances neuroprotection following intracerebral hemorrhage via the miR-326-mediated autophagy. Stem Cell Res Ther. 2021; 12: 413.

60. Deng L, Zhou L, Zhu Y, Fan G, Tang H, Zheng Y, Gao X, Guo K, Zhou P, Yang C. Electroacupuncture Enhance Therapeutic Efficacy of Mesenchymal Stem Cells Transplantation in Rats With Intracerebral Hemorrhage. Stem Cell Rev Rep. 2021.

61. Tang B, Song M, Xie X, Le D, Tu Q, Wu X, Chen M. Tumor Necrosis Factor-stimulated Gene-6 (TSG-6) Secreted by BMSCs Regulates Activated Astrocytes by Inhibiting NF-κB Signaling Pathway to Ameliorate Blood Brain Barrier Damage After Intracerebral Hemorrhage. Neurochem Res. 2021; 46: 2387-402.

62. Yip HK, Lin KC, Sung PH, Chiang JY, Yin TC, Wu RW, Chen KH. Umbilical cord-derived MSC and hyperbaric oxygen therapy effectively protected the brain in rat after acute intracerebral haemorrhage. J Cell Mol Med. 2021; 25: 5640-54.
