# Supplementary material for: Efficacy of stem cell therapy in animal models of intracerebral hemorrhage: an updated meta-analysis
Source: Stem Cell Res Ther. 2022 Sep 5;13:452. doi: 10.1186/s13287-022-03158-7 (PMC9446670; doi:10.1186/s13287-022-03158-7)

eFig1. Subgroup analysis by species for tissue loss.

Group by species	Study name	Statistics for each study			
		Std diff in means	Lower limit	Upper limit	p-Value
mice	Wakai,2014	-2.159	-3.478	-0.841	0.001
mice	Wakai,2016	-1.311	-2.558	-0.064	0.039
mice		-1.727	-3.277	-0.177	0.029
rats	Jeong,2003	-1.433	-2.312	-0.553	0.001
rats	Seyfried-1,2006	-3.479	-4.905	-2.052	0.000
rats	Seyfried-2,2006	-3.769	-5.354	-2.184	0.000
rats	Seyfried-3,2006	-2.519	-3.757	-1.282	0.000
rats	Kim,2007	-1.995	-3.691	-0.299	0.021
rats	Fatar,2008	0.433	-0.454	1.320	0.339
rats	Seyfried,2008	-0.913	-1.884	0.058	0.065
rats	Liao,2009	-1.817	-3.291	-0.344	0.016
rats	Seyfried-1,2010	-0.230	-1.157	0.697	0.627
rats	Seyfried-2,2010	-3.663	-5.175	-2.151	0.000
rats	Yang CX,2011	-2.344	-3.954	-0.734	0.004
rats	Yang DM-1,2012	-0.877	-1.903	0.149	0.094
rats	Yang DM-2,2012	-0.849	-1.872	0.174	0.104
rats	Yang DM-3,2012	-1.072	-2.120	-0.024	0.045
rats	Liang,2013	-0.241	-1.225	0.742	0.631
rats	Seghatoleslam-1,2013	-0.390	-1.275	0.495	0.388
rats	Seghatoleslam-2,2013	-0.549	-1.442	0.344	0.228
rats	Seghatoleslam-3,2013	-0.909	-1.830	0.011	0.053
rats	Kim,2015	-1.430	-2.471	-0.390	0.007
rats	Suda-1,2015	-1.495	-2.680	-0.310	0.013
rats	Suda-2,2015	-1.236	-2.380	-0.093	0.034
rats	Xie-1,2016	-2.421	-4.052	-0.789	0.004
rats	Xie-2,2016	-2.313	-3.915	-0.712	0.005
rats	Huang-1,2019	-3.336	-5.480	-1.193	0.002
rats	Huang-2,2019	-2.134	-3.870	-0.398	0.016
rats	Huang-3,2019	-3.986	-6.380	-1.591	0.001
rats	Mello-1,2020	-0.141	-1.043	0.761	0.759
rats	Mello-2,2020	0.183	-0.590	0.956	0.642
rats		-1.398	-1.808	-0.988	0.000

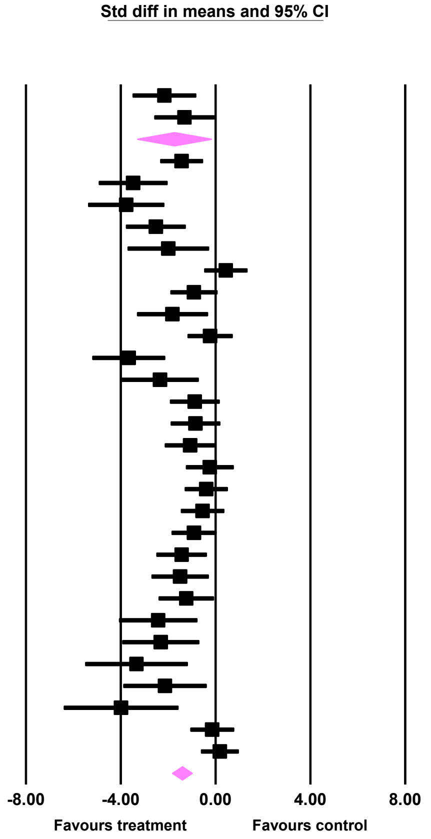

eFig2. Subgroup analysis by quality for tissue loss.

Group by quality	Study name	Std diff in means	Lower limit	Upper limit	p-Value
high	Jeong,2003	-1.433	-2.312	-0.553	0.001
high	Liao,2009	-1.817	-3.291	-0.344	0.016
high	Yang CX,2011	-2.344	-3.954	-0.734	0.004
high	Liang,2013	-0.241	-1.225	0.742	0.631
high	Seghatoleslam-1,2013	-0.390	-1.275	0.495	0.388
high	Seghatoleslam-2,2013	-0.549	-1.442	0.344	0.228
high	Seghatoleslam-3,2013	-0.909	-1.830	0.011	0.053
high	Wakai,2014	-2.159	-3.478	-0.841	0.001
high	Suda-1,2015	-1.495	-2.680	-0.310	0.013
high	Suda-2,2015	-1.236	-2.380	-0.093	0.034
high	Xie-1,2016	-2.421	-4.052	-0.789	0.004
high	Xie-2,2016	-2.313	-3.915	-0.712	0.005
high	Wakai,2016	-1.311	-2.558	-0.064	0.039
high	Mello-1,2020	-0.141	-1.043	0.761	0.759
high	Mello-2,2020	0.183	-0.590	0.956	0.642
high		-1.127	-1.669	-0.584	0.000
low	Seyfried-1,2006	-3.479	-4.905	-2.052	0.000
low	Seyfried-2,2006	-3.769	-5.354	-2.184	0.000
low	Seyfried-3,2006	-2.519	-3.757	-1.282	0.000
low	Kim,2007	-1.995	-3.691	-0.299	0.021
low	Fatar,2008	0.433	-0.454	1.320	0.339
low	Seyfried,2008	-0.913	-1.884	0.058	0.065
low	Seyfried-1,2010	-0.230	-1.157	0.697	0.627
low	Seyfried-2,2010	-3.663	-5.175	-2.151	0.000
low	Yang DM-1,2012	-0.877	-1.903	0.149	0.094
low	Yang DM-2,2012	-0.849	-1.872	0.174	0.104
low	Yang DM-3,2012	-1.072	-2.120	-0.024	0.045
low	Kim,2015	-1.430	-2.471	-0.390	0.007
low	Huang-1,2019	-3.336	-5.480	-1.193	0.002
low	Huang-2,2019	-2.134	-3.870	-0.398	0.016
low	Huang-3,2019	-3.986	-6.380	-1.591	0.001
low		-1.737	-2.307	-1.167	0.000

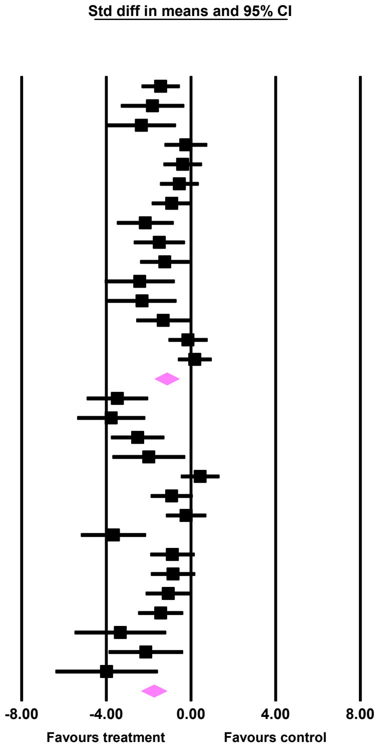

eFig3. Subgroup analysis by methods of ICH for tissue loss.

Group by methods of ICH	Study name	Std diff in means	Lower limit	Upper limit	p-Value
autologous blood	Seyfried-1,2006	-3.479	-4.905	-2.052	0.000
autologous blood	Seyfried-2,2006	-3.769	-5.354	-2.184	0.000
autologous blood	Seyfried-3,2006	-2.519	-3.757	-1.282	0.000
autologous blood	Seyfried,2008	-0.913	-1.884	0.058	0.065
autologous blood	Seyfried-1,2010	-0.230	-1.157	0.697	0.627
autologous blood	Seyfried-2,2010	-3.663	-5.175	-2.151	0.000
autologous blood	Yang DM-1,2012	-0.877	-1.903	0.149	0.094
autologous blood	Yang DM-2,2012	-0.849	-1.872	0.174	0.104
autologous blood	Yang DM-3,2012	-1.072	-2.120	-0.024	0.045
autologous blood	Wakai,2014	-2.159	-3.478	-0.841	0.001
autologous blood	Suda-1,2015	-1.495	-2.680	-0.310	0.013
autologous blood	Suda-2,2015	-1.236	-2.380	-0.093	0.034
autologous blood	Wakai,2016	-1.311	-2.558	-0.064	0.039
autologous blood	Huang-1,2019	-3.336	-5.480	-1.193	0.002
autologous blood	Huang-2,2019	-2.134	-3.870	-0.398	0.016
autologous blood	Huang-3,2019	-3.986	-6.380	-1.591	0.001
autologous blood		-1.841	-2.358	-1.325	0.000
collagenase	Jeong,2003	-1.433	-2.312	-0.553	0.001
collagenase	Kim,2007	-1.995	-3.691	-0.299	0.021
collagenase	Fatar,2008	0.433	-0.454	1.320	0.339
collagenase	Liao,2009	-1.817	-3.291	-0.344	0.016
collagenase	Yang CX,2011	-2.344	-3.954	-0.734	0.004
collagenase	Liang,2013	-0.241	-1.225	0.742	0.631
collagenase	Seghatoleslam-1,2013	-0.390	-1.275	0.495	0.388
collagenase	Seghatoleslam-2,2013	-0.549	-1.442	0.344	0.228
collagenase	Seghatoleslam-3,2013	-0.909	-1.830	0.011	0.053
collagenase	Kim,2015	-1.430	-2.471	-0.390	0.007
collagenase	Xie-1,2016	-2.421	-4.052	-0.789	0.004
collagenase	Xie-2,2016	-2.313	-3.915	-0.712	0.005
collagenase	Mello-1,2020	-0.141	-1.043	0.761	0.759
collagenase	Mello-2,2020	0.183	-0.590	0.956	0.642
collagenase		-0.936	-1.456	-0.416	0.000

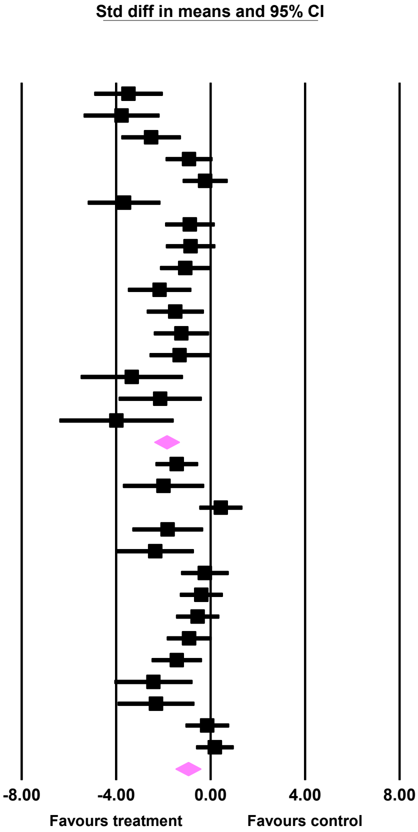

eFig4. Subgroup analysis by delivery routes for tissue loss

Group by delivery routes	Study name	Std diff in means	Lower limit	Upper limit	p-Value
intra-arterial	Seyfried,2008	-0.913	-1.884	0.058	0.065
intra-arterial		-0.913	-2.926	1.100	0.374
intracerebral	Liao,2009	-1.817	-3.291	-0.344	0.016
intracerebral	Yang CX,2011	-2.344	-3.954	-0.734	0.004
intracerebral	Liang,2013	-0.241	-1.225	0.742	0.631
intracerebral	Wakai,2014	-2.159	-3.478	-0.841	0.001
intracerebral	Kim,2015	-1.430	-2.471	-0.390	0.007
intracerebral	Xie-1,2016	-2.421	-4.052	-0.789	0.004
intracerebral	Wakai,2016	-1.311	-2.558	-0.064	0.039
intracerebral	Huang-1,2019	-3.336	-5.480	-1.193	0.002
intracerebral	Huang-2,2019	-2.134	-3.870	-0.398	0.016
intracerebral	Huang-3,2019	-3.986	-6.380	-1.591	0.001
intracerebral		-1.912	-2.647	-1.176	0.000
intravenous	Jeong,2003	-1.433	-2.312	-0.553	0.001
intravenous	Seyfried-1,2006	-3.479	-4.905	-2.052	0.000
intravenous	Seyfried-2,2006	-3.769	-5.354	-2.184	0.000
intravenous	Seyfried-3,2006	-2.519	-3.757	-1.282	0.000
intravenous	Kim,2007	-1.995	-3.691	-0.299	0.021
intravenous	Fatar,2008	0.433	-0.454	1.320	0.339
intravenous	Seyfried-1,2010	-0.230	-1.157	0.697	0.627
intravenous	Seyfried-2,2010	-3.663	-5.175	-2.151	0.000
intravenous	Yang DM-1,2012	-0.877	-1.903	0.149	0.094
intravenous	Yang DM-2,2012	-0.849	-1.872	0.174	0.104
intravenous	Yang DM-3,2012	-1.072	-2.120	-0.024	0.045
intravenous	Seghatoleslam-1,2013	-0.390	-1.275	0.495	0.388
intravenous	Seghatoleslam-2,2013	-0.549	-1.442	0.344	0.228
intravenous	Seghatoleslam-3,2013	-0.909	-1.830	0.011	0.053
intravenous	Suda-1,2015	-1.495	-2.680	-0.310	0.013
intravenous	Suda-2,2015	-1.236	-2.380	-0.093	0.034
intravenous	Xie-2,2016	-2.313	-3.915	-0.712	0.005
intravenous	Mello-1,2020	-0.141	-1.043	0.761	0.759
intravenous	Mello-2,2020	0.183	-0.590	0.956	0.642
intravenous		-1.236	-1.716	-0.756	0.000

Statistics for each study

Std diff in means and 95% CI

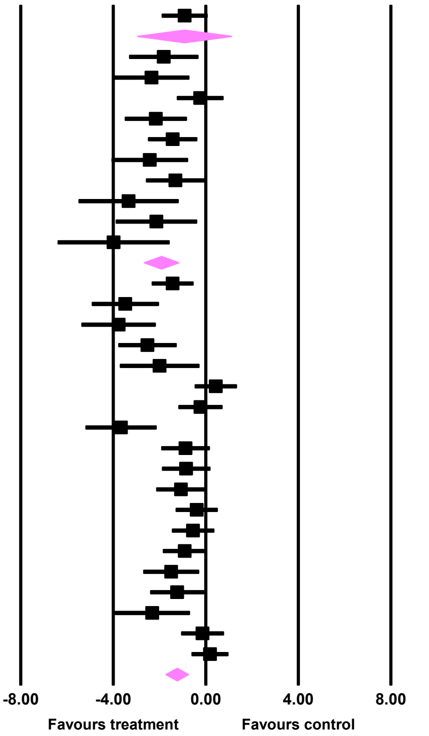

eFig5. Subgroup analysis by sources of stem cells for tissue loss

Group by sources of stem cells	Study name	Std diff in means	Lower limit	Upper limit	p-Value
allogenic	Seyfried-1,2010	-0.230	-1.157	0.697	0.627
allogenic	Seyfried-2,2010	-3.663	-5.175	-2.151	0.000
allogenic	Yang CX,2011	-2.344	-3.954	-0.734	0.004
allogenic	Liang,2013	-0.241	-1.225	0.742	0.631
allogenic	Wakai,2014	-2.159	-3.478	-0.841	0.001
allogenic	Suda-1,2015	-1.495	-2.680	-0.310	0.013
allogenic	Suda-2,2015	-1.236	-2.380	-0.093	0.034
allogenic	Wakai,2016	-1.311	-2.558	-0.064	0.039
allogenic		-1.477	-2.250	-0.704	0.000
xenogenic	Jeong,2003	-1.433	-2.312	-0.553	0.001
xenogenic	Seyfried-1,2006	-3.479	-4.905	-2.052	0.000
xenogenic	Seyfried-2,2006	-3.769	-5.354	-2.184	0.000
xenogenic	Seyfried-3,2006	-2.519	-3.757	-1.282	0.000
xenogenic	Kim,2007	-1.995	-3.691	-0.299	0.021
xenogenic	Fatar,2008	0.433	-0.454	1.320	0.339
xenogenic	Seyfried,2008	-0.913	-1.884	0.058	0.065
xenogenic	Liao,2009	-1.817	-3.291	-0.344	0.016
xenogenic	Yang DM-1,2012	-0.877	-1.903	0.149	0.094
xenogenic	Yang DM-2,2012	-0.849	-1.872	0.174	0.104
xenogenic	Yang DM-3,2012	-1.072	-2.120	-0.024	0.045
xenogenic	Seghatoleslam-1,2013	-0.390	-1.275	0.495	0.388
xenogenic	Seghatoleslam-2,2013	-0.549	-1.442	0.344	0.228
xenogenic	Seghatoleslam-3,2013	-0.909	-1.830	0.011	0.053
xenogenic	Kim,2015	-1.430	-2.471	-0.390	0.007
xenogenic	Xie-1,2016	-2.421	-4.052	-0.789	0.004
xenogenic	Xie-2,2016	-2.313	-3.915	-0.712	0.005
xenogenic	Huang-1,2019	-3.336	-5.480	-1.193	0.002
xenogenic	Huang-2,2019	-2.134	-3.870	-0.398	0.016
xenogenic	Huang-3,2019	-3.986	-6.380	-1.591	0.001
xenogenic	Mello-1,2020	-0.141	-1.043	0.761	0.759
xenogenic	Mello-2,2020	0.183	-0.590	0.956	0.642
xenogenic		-1.403	-1.871	-0.935	0.000

Std diff in means and 95% CI

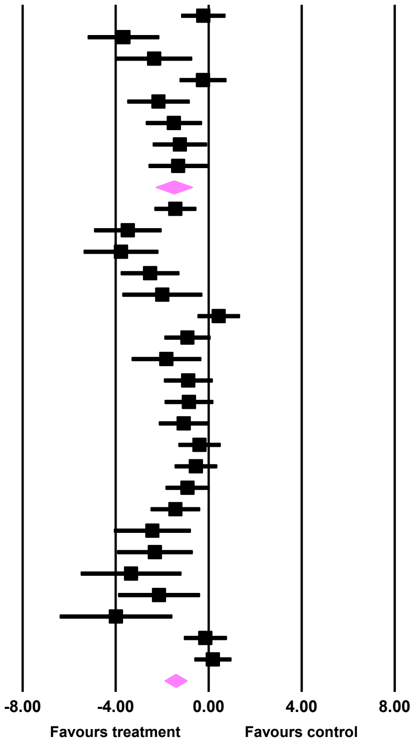

eFig6. Subgroup analysis by types of stem cells for tissue loss

Group by types of stem cells	Study name	Std diff in means	Lower limit	Upper limit	p-Value
MNCs	Seghatoleslam-1,2013	-0.390	-1.275	0.495	0.388
MNCs	Seghatoleslam-2,2013	-0.549	-1.442	0.344	0.228
MNCs	Seghatoleslam-3,2013	-0.909	-1.830	0.011	0.053
MNCs	Suda-1,2015	-1.495	-2.680	-0.310	0.013
MNCs	Suda-2,2015	-1.236	-2.380	-0.093	0.034
MNCs		-0.893	-1.825	0.040	0.061
MSCs	Seyfried-1,2006	-3.479	-4.905	-2.052	0.000
MSCs	Seyfried-2,2006	-3.769	-5.354	-2.184	0.000
MSCs	Seyfried-3,2006	-2.519	-3.757	-1.282	0.000
MSCs	Kim,2007	-1.995	-3.691	-0.299	0.021
MSCs	Fatar,2008	0.433	-0.454	1.320	0.339
MSCs	Seyfried,2008	-0.913	-1.884	0.058	0.065
MSCs	Liao,2009	-1.817	-3.291	-0.344	0.016
MSCs	Seyfried-1,2010	-0.230	-1.157	0.697	0.627
MSCs	Seyfried-2,2010	-3.663	-5.175	-2.151	0.000
MSCs	Yang CX,2011	-2.344	-3.954	-0.734	0.004
MSCs	Yang DM-1,2012	-0.877	-1.903	0.149	0.094
MSCs	Yang DM-2,2012	-0.849	-1.872	0.174	0.104
MSCs	Yang DM-3,2012	-1.072	-2.120	-0.024	0.045
MSCs	Liang,2013	-0.241	-1.225	0.742	0.631
MSCs	Kim,2015	-1.430	-2.471	-0.390	0.007
MSCs	Xie-1,2016	-2.421	-4.052	-0.789	0.004
MSCs	Xie-2,2016	-2.313	-3.915	-0.712	0.005
MSCs	Huang-1,2019	-3.336	-5.480	-1.193	0.002
MSCs	Huang-2,2019	-2.134	-3.870	-0.398	0.016
MSCs	Huang-3,2019	-3.986	-6.380	-1.591	0.001
MSCs	Mello-1,2020	-0.141	-1.043	0.761	0.759
MSCs	Mello-2,2020	0.183	-0.590	0.956	0.642
MSCs		-1.539	-2.019	-1.058	0.000
NSCs	Jeong,2003	-1.433	-2.312	-0.553	0.001
NSCs	Wakai,2014	-2.159	-3.478	-0.841	0.001
NSCs	Wakai,2016	-1.311	-2.558	-0.064	0.039
NSCs		-1.616	-2.862	-0.371	0.011

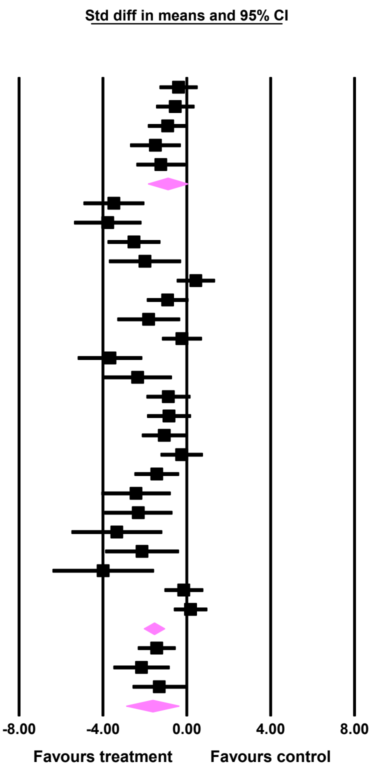

eFig7. Subgroup analysis by time administration of stem cells for tissue loss

Group by time administration	Study name	Std diff in means	Lower limit	Upper limit	p-Value
[0h, 8h]	Xie-1,2016	-2.421	-4.052	-0.789	0.004
[0h, 8h]	Xie-2,2016	-2.313	-3.915	-0.712	0.005
[0h, 8h]	Huang-1,2019	-3.336	-5.480	-1.193	0.002
[0h, 8h]	Huang-2,2019	-2.134	-3.870	-0.398	0.016
[0h, 8h]	Huang-3,2019	-3.986	-6.380	-1.591	0.001
[0h, 8h]		-2.716	-3.814	-1.618	0.000
>24h	Yang CX,2011	-2.344	-3.954	-0.734	0.004
>24h	Yang DM-2,2012	-0.849	-1.872	0.174	0.104
>24h	Yang DM-3,2012	-1.072	-2.120	-0.024	0.045
>24h	Wakai,2014	-2.159	-3.478	-0.841	0.001
>24h	Kim,2015	-1.430	-2.471	-0.390	0.007
>24h	Wakai,2016	-1.311	-2.558	-0.064	0.039
>24h		-1.469	-2.285	-0.654	0.000
24 h	Jeong,2003	-1.433	-2.312	-0.553	0.001
24 h	Seyfried-1,2006	-3.479	-4.905	-2.052	0.000
24 h	Seyfried-2,2006	-3.769	-5.354	-2.184	0.000
24 h	Seyfried-3,2006	-2.519	-3.757	-1.282	0.000
24 h	Kim,2007	-1.995	-3.691	-0.299	0.021
24 h	Fatar,2008	0.433	-0.454	1.320	0.339
24 h	Seyfried,2008	-0.913	-1.884	0.058	0.065
24 h	Liao,2009	-1.817	-3.291	-0.344	0.016
24 h	Seyfried-1,2010	-0.230	-1.157	0.697	0.627
24 h	Seyfried-2,2010	-3.663	-5.175	-2.151	0.000
24 h	Yang DM-1,2012	-0.877	-1.903	0.149	0.094
24 h	Liang,2013	-0.241	-1.225	0.742	0.631
24 h	Seghatoleslam-1,2013	-0.390	-1.275	0.495	0.388
24 h	Seghatoleslam-2,2013	-0.549	-1.442	0.344	0.228
24 h	Seghatoleslam-3,2013	-0.909	-1.830	0.011	0.053
24 h	Suda-1,2015	-1.495	-2.680	-0.310	0.013
24 h	Suda-2,2015	-1.236	-2.380	-0.093	0.034
24 h	Mello-1,2020	-0.141	-1.043	0.761	0.759
24 h	Mello-2,2020	0.183	-0.590	0.956	0.642
24 h		-1.154	-1.600	-0.709	0.000

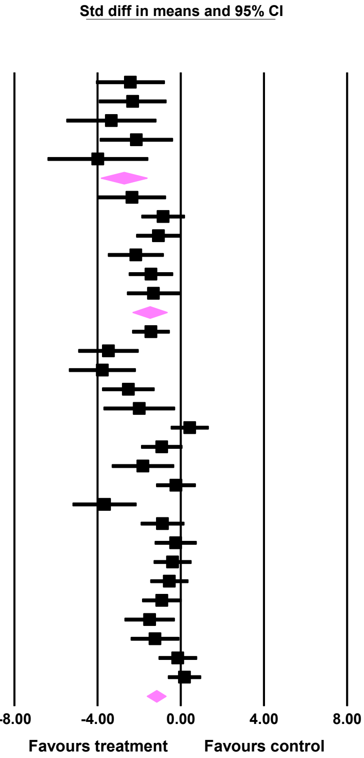

eFig7. Subgroup analysis by doses of stem cells for tissue loss

Group by cell doses	Study name	Std diff in means	Lower limit	Upper limit	p-Value
[1-5]*106	Jeong,2003	-1.433	-2.312	-0.553	0.001
[1-5]*106	Seyfried-1,2006	-3.479	-4.905	-2.052	0.000
[1-5]*106	Seyfried-2,2006	-3.769	-5.354	-2.184	0.000
[1-5]*106	Kim,2007	-1.995	-3.691	-0.299	0.021
[1-5]*106	Fatar,2008	0.433	-0.454	1.320	0.339
[1-5]*106	Seyfried,2008	-0.913	-1.884	0.058	0.065
[1-5]*106	Seyfried-2,2010	-3.663	-5.175	-2.151	0.000
[1-5]*106	Yang DM-1,2012	-0.877	-1.903	0.149	0.094
[1-5]*106	Yang DM-2,2012	-0.849	-1.872	0.174	0.104
[1-5]*106	Yang DM-3,2012	-1.072	-2.120	-0.024	0.045
[1-5]*106	Liang,2013	-0.241	-1.225	0.742	0.631
[1-5]*106	Seghatoleslam-1,2013	-0.390	-1.275	0.495	0.388
[1-5]*106	Suda-1,2015	-1.495	-2.680	-0.310	0.013
[1-5]*106	Suda-2,2015	-1.236	-2.380	-0.093	0.034
[1-5]*106	Xie-2,2016	-2.313	-3.915	-0.712	0.005
[1-5]*106	Huang-3,2019	-3.986	-6.380	-1.591	0.001
[1-5]*106	Mello-1,2020	-0.141	-1.043	0.761	0.759
[1-5]*106	Mello-2,2020	0.183	-0.590	0.956	0.642
[1-5]*106		-1.298	-1.808	-0.787	0.000
< 1*106	Liao,2009	-1.817	-3.291	-0.344	0.016
< 1*106	Seyfried-1,2010	-0.230	-1.157	0.697	0.627
< 1*106	Yang CX,2011	-2.344	-3.954	-0.734	0.004
< 1*106	Wakai,2014	-2.159	-3.478	-0.841	0.001
< 1*106	Kim,2015	-1.430	-2.471	-0.390	0.007
< 1*106	Xie-1,2016	-2.421	-4.052	-0.789	0.004
< 1*106	Wakai,2016	-1.311	-2.558	-0.064	0.039
< 1*106	Huang-1,2019	-3.336	-5.480	-1.193	0.002
< 1*106	Huang-2,2019	-2.134	-3.870	-0.398	0.016
< 1*106		-1.773	-2.541	-1.004	0.000
> 5*106	Seyfried-3,2006	-2.519	-3.757	-1.282	0.000
> 5*106	Seghatoleslam-2,2013	-0.549	-1.442	0.344	0.228
> 5*106	Seghatoleslam-3,2013	-0.909	-1.830	0.011	0.053
> 5*106		-1.263	-2.459	-0.068	0.038

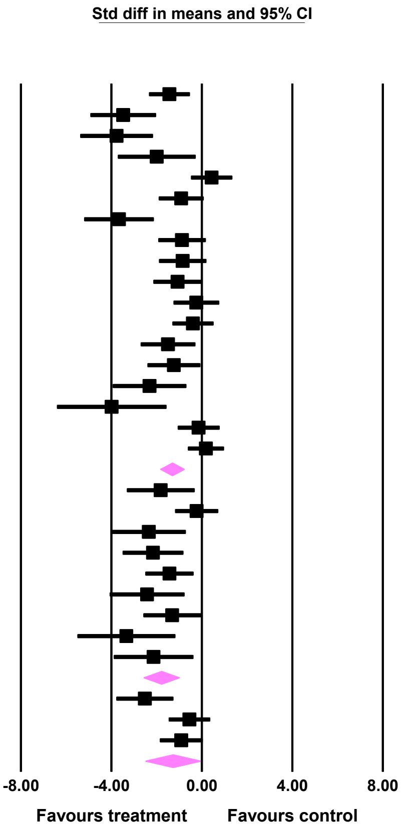

Supplement: Supplementary file 3 — Additional file 3. Funnel plot of sensitivity analysis for mNSS. [file 13287_2022_3158_MOESM3_ESM.pdf]
