# Supplementary material for: Efficacy of stem cell therapy in animal models of intracerebral hemorrhage: an updated meta-analysis
Source: Stem Cell Res Ther. 2022 Sep 5;13:452. doi: 10.1186/s13287-022-03158-7 (PMC9446670; doi:10.1186/s13287-022-03158-7)

eFig1. Subgroup analysis by species for mNSS.

Effects of stem cells on mNSS

Group by species	author	Std diff in means	Lower limit	Upper limit	p-Value
mice	Kuramoto,2019	-0.50	-1.37	0.37	0.263
mice	Zhang Y,2019	-1.17	-2.39	0.05	0.061
mice	Chen X,2020	-7.20	-9.60	-4.80	0.000
mice	Gao,2020	-3.61	-5.44	-1.77	0.000
mice	Liu-1,2021	-1.41	-2.79	-0.02	0.046
mice	Liu-2,2021	-1.98	-3.50	-0.47	0.010
mice	Hu-1,2021	-5.23	-7.08	-3.39	0.000
mice	Hu-2,2021	-4.11	-5.66	-2.56	0.000
mice		-2.81	-3.79	-1.84	0.000
monkeys	Feng-1,2011	-2.87	-4.52	-1.21	0.001
monkeys	Feng-2,2011	-2.89	-4.56	-1.23	0.001
monkeys		-2.88	-4.87	-0.89	0.005
rats	Seyfried-1,2006	-1.10	-2.07	-0.13	0.026
rats	Seyfried-2,2006	-1.42	-2.49	-0.36	0.009
rats	Seyfried-3,2006	-1.26	-2.27	-0.24	0.015
rats	Zhang-1,2006	-1.39	-2.08	-0.70	0.000
rats	Zhang-2,2006	-0.15	-0.77	0.47	0.630
rats	Zhang-3,2006	-1.69	-2.41	-0.97	0.000
rats	Li F-1,2007	-1.83	-3.00	-0.66	0.002
rats	Li F-2,2007	-7.06	-9.70	-4.43	0.000
rats	Li F-3,2007	-5.19	-7.24	-3.14	0.000
rats	Li F-4,2007	-0.74	-1.75	0.27	0.152
rats	Li F-5,2007	-0.44	-1.43	0.55	0.388
rats	Seyfried,2008	-0.90	-1.87	0.07	0.068
rats	Liao,2009	-2.05	-3.29	-0.82	0.001
rats	Otero,2010	-6.82	-9.11	-4.53	0.000
rats	Seyfried-1,2010	-0.71	-1.67	0.24	0.143
rats	Seyfried-2,2010	-1.34	-2.36	-0.32	0.010
rats	Tang,2010	-2.88	-3.87	-1.89	0.000
rats	Otero,2011	-7.74	-10.30	-5.19	0.000
rats	Yang CX,2011	-1.99	-3.51	-0.48	0.010
rats	Yang DM-1,2012	-2.41	-3.70	-1.12	0.000
rats	Yang DM-2,2012	-1.61	-2.74	-0.48	0.005
rats	Yang DM-3,2012	-1.13	-2.19	-0.08	0.035
rats	Chen J,2012	-2.93	-4.71	-1.14	0.001
rats	Wang SP,2012	-4.23	-6.27	-2.20	0.000
rats	Yang KL,2013	-1.83	-3.18	-0.49	0.008
rats	Bao,2013	-2.34	-3.37	-1.30	0.000
rats	Qin,2013	-3.83	-5.18	-2.48	0.000
rats	Gao,2014	-2.60	-4.14	-1.07	0.001
rats	Wang CY,2015	-2.85	-3.66	-2.05	0.000
rats	Chen M,2015	-4.04	-6.02	-2.07	0.000
rats	Zhang,2015	-1.82	-3.16	-0.47	0.008
rats	Xie-1,2016	-0.86	-2.05	0.32	0.152
rats	Xie-2,2016	-0.91	-2.10	0.28	0.133
rats	Zhou,2016	-2.03	-3.42	-0.63	0.004
rats	Cui CM,2017	-1.87	-3.36	-0.39	0.014
rats	Ding,2017	-3.53	-5.34	-1.72	0.000
rats	Zhang,2017	-1.85	-2.89	-0.80	0.001
rats	Deng,2021	-2.04	-3.57	-0.51	0.009
rats	Tang,2021	-2.17	-3.59	-0.74	0.003
rats		-2.14	-2.56	-1.72	0.000

Std diff in means and 95% CI

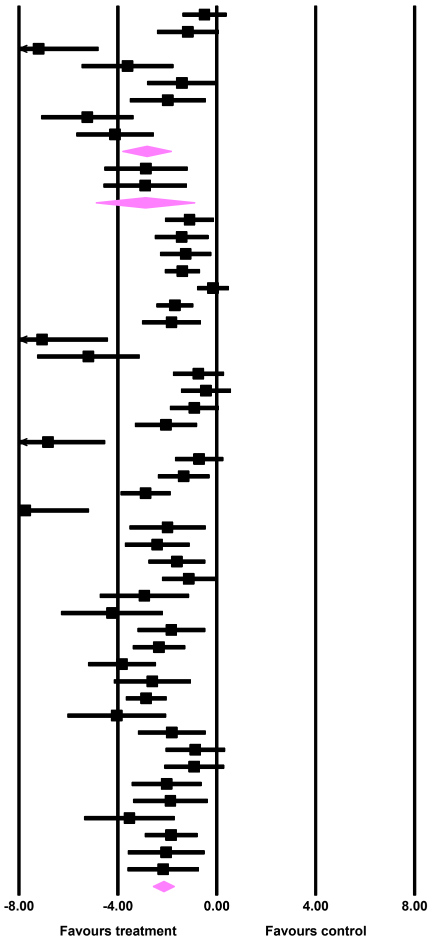

eFig2. Subgroup analysis by quality for mNSS.

Effects of stem cells on mNSS

Group by quality	author	Std diff in means	Lower limit	Upper limit	p-Value
high	Li F-1,2007	-1.83	-3.00	-0.66	0.002
high	Li F-2,2007	-7.06	-9.70	-4.43	0.000
high	Li F-3,2007	-5.19	-7.24	-3.14	0.000
high	Li F-4,2007	-0.74	-1.75	0.27	0.152
high	Li F-5,2007	-0.44	-1.43	0.55	0.388
high	Liao,2009	-2.05	-3.29	-0.82	0.001
high	Feng-1,2011	-2.87	-4.52	-1.21	0.001
high	Feng-2,2011	-2.89	-4.56	-1.23	0.001
high	Otero,2011	-7.74	-10.30	-5.19	0.000
high	Yang CX,2011	-1.99	-3.51	-0.48	0.010
high	Chen J,2012	-2.93	-4.71	-1.14	0.001
high	Wang SP,2012	-4.23	-6.27	-2.20	0.000
high	Yang KL,2013	-1.83	-3.18	-0.49	0.008
high	Bao,2013	-2.34	-3.37	-1.30	0.000
high	Qin,2013	-3.83	-5.18	-2.48	0.000
high	Gao,2014	-2.60	-4.14	-1.07	0.001
high	Wang CY,2015	-2.85	-3.66	-2.05	0.000
high	Chen M,2015	-4.04	-6.02	-2.07	0.000
high	Zhang,2015	-1.82	-3.16	-0.47	0.008
high	Xie-1,2016	-0.86	-2.05	0.32	0.152
high	Xie-2,2016	-0.91	-2.10	0.28	0.133
high	Zhou,2016	-2.03	-3.42	-0.63	0.004
high	Cui CM,2017	-1.87	-3.36	-0.39	0.014
high	Ding,2017	-3.53	-5.34	-1.72	0.000
high	Zhang,2017	-1.85	-2.89	-0.80	0.001
high	Kuramoto,2019	-0.50	-1.37	0.37	0.263
high	Zhang Y,2019	-1.17	-2.39	0.05	0.061
high	Chen X,2020	-7.20	-9.60	-4.80	0.000
high	Gao,2020	-3.61	-5.44	-1.77	0.000
high	Liu-1,2021	-1.41	-2.79	-0.02	0.046
high	Liu-2,2021	-1.98	-3.50	-0.47	0.010
high	Hu-1,2021	-5.23	-7.08	-3.39	0.000
high	Hu-2,2021	-4.11	-5.66	-2.56	0.000
high	Deng,2021	-2.04	-3.57	-0.51	0.009
high	Tang,2021	-2.17	-3.59	-0.74	0.003
high		-2.57	-3.01	-2.13	0.000
low	Seyfried-1,2006	-1.10	-2.07	-0.13	0.026
low	Seyfried-2,2006	-1.42	-2.49	-0.36	0.009
low	Seyfried-3,2006	-1.26	-2.27	-0.24	0.015
low	Zhang-1,2006	-1.39	-2.08	-0.70	0.000
low	Zhang-2,2006	-0.15	-0.77	0.47	0.630
low	Zhang-3,2006	-1.69	-2.41	-0.97	0.000
low	Seyfried,2008	-0.90	-1.87	0.07	0.068
low	Otero,2010	-6.82	-9.11	-4.53	0.000
low	Seyfried-1,2010	-0.71	-1.67	0.24	0.143
low	Seyfried-2,2010	-1.34	-2.36	-0.32	0.010
low	Tang,2010	-2.88	-3.87	-1.89	0.000
low	Yang DM-1,2012	-2.41	-3.70	-1.12	0.000
low	Yang DM-2,2012	-1.61	-2.74	-0.48	0.005
low	Yang DM-3,2012	-1.13	-2.19	-0.08	0.035
low		-1.59	-2.22	-0.96	0.000

Std diff in means and 95% CI

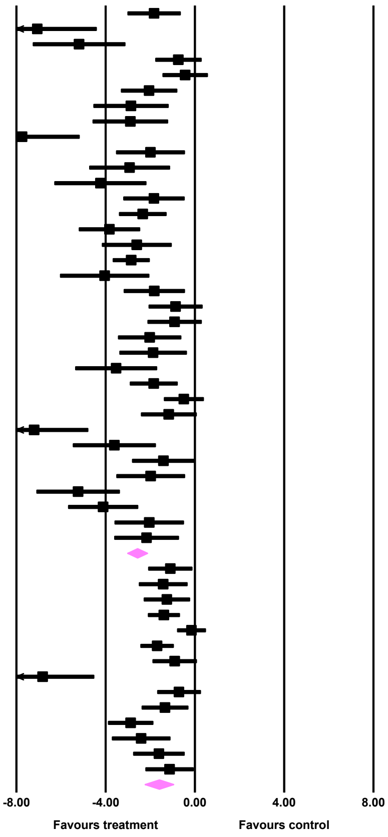

eFig3. Subgroup analysis by methods of ICH for mNSS

Effects of stem cells on mNSS

Group by methods of ICH	author	Std diff in means	Lower limit	Upper limit	p-Value
		Statistics for each study			
autologous blood	Seyfried-1,2006	-1.10	-2.07	-0.13	0.026
autologous blood	Seyfried-2,2006	-1.42	-2.49	-0.36	0.009
autologous blood	Seyfried-3,2006	-1.26	-2.27	-0.24	0.015
autologous blood	Seyfried,2008	-0.90	-1.87	0.07	0.068
autologous blood	Seyfried-1,2010	-0.71	-1.67	0.24	0.143
autologous blood	Seyfried-2,2010	-1.34	-2.36	-0.32	0.010
autologous blood	Feng-1,2011	-2.87	-4.52	-1.21	0.001
autologous blood	Feng-2,2011	-2.89	-4.56	-1.23	0.001
autologous blood	Yang DM-1,2012	-2.41	-3.70	-1.12	0.000
autologous blood	Yang DM-2,2012	-1.61	-2.74	-0.48	0.005
autologous blood	Yang DM-3,2012	-1.13	-2.19	-0.08	0.035
autologous blood	Wang CY,2015	-2.85	-3.66	-2.05	0.000
autologous blood	Cui CM,2017	-1.87	-3.36	-0.39	0.014
autologous blood	Gao,2020	-3.61	-5.44	-1.77	0.000
autologous blood		-1.78	-2.47	-1.09	0.000
collagenase	Zhang-1,2006	-1.39	-2.08	-0.70	0.000
collagenase	Zhang-2,2006	-0.15	-0.77	0.47	0.630
collagenase	Zhang-3,2006	-1.69	-2.41	-0.97	0.000
collagenase	Li F-1,2007	-1.83	-3.00	-0.66	0.002
collagenase	Li F-2,2007	-7.06	-9.70	-4.43	0.000
collagenase	Li F-3,2007	-5.19	-7.24	-3.14	0.000
collagenase	Li F-4,2007	-0.74	-1.75	0.27	0.152
collagenase	Li F-5,2007	-0.44	-1.43	0.55	0.388
collagenase	Liao,2009	-2.05	-3.29	-0.82	0.001
collagenase	Otero,2010	-6.82	-9.11	-4.53	0.000
collagenase	Tang,2010	-2.88	-3.87	-1.89	0.000
collagenase	Otero,2011	-7.74	-10.30	-5.19	0.000
collagenase	Yang CX,2011	-1.99	-3.51	-0.48	0.010
collagenase	Chen J,2012	-2.93	-4.71	-1.14	0.001
collagenase	Wang SP,2012	-4.23	-6.27	-2.20	0.000
collagenase	Yang KL,2013	-1.83	-3.18	-0.49	0.008
collagenase	Bao,2013	-2.34	-3.37	-1.30	0.000
collagenase	Qin,2013	-3.83	-5.18	-2.48	0.000
collagenase	Gao,2014	-2.60	-4.14	-1.07	0.001
collagenase	Chen M,2015	-4.04	-6.02	-2.07	0.000
collagenase	Zhang,2015	-1.82	-3.16	-0.47	0.008
collagenase	Xie-1,2016	-0.86	-2.05	0.32	0.152
collagenase	Xie-2,2016	-0.91	-2.10	0.28	0.133
collagenase	Zhou,2016	-2.03	-3.42	-0.63	0.004
collagenase	Zhang,2017	-1.85	-2.89	-0.80	0.001
collagenase	Kuramoto,2019	-0.50	-1.37	0.37	0.263
collagenase	Zhang Y,2019	-1.17	-2.39	0.05	0.061
collagenase	Chen X,2020	-7.20	-9.60	-4.80	0.000
collagenase	Liu-1,2021	-1.41	-2.79	-0.02	0.046
collagenase	Liu-2,2021	-1.98	-3.50	-0.47	0.010
collagenase	Hu-1,2021	-5.23	-7.08	-3.39	0.000
collagenase	Hu-2,2021	-4.11	-5.66	-2.56	0.000
collagenase	Deng,2021	-2.04	-3.57	-0.51	0.009
collagenase	Tang,2021	-2.17	-3.59	-0.74	0.003
collagenase		-2.46	-2.92	-2.00	0.000
hemoglobin	Ding,2017	-3.53	-5.34	-1.72	0.000
hemoglobin		-3.53	-6.44	-0.62	0.018

Std diff in means and 95% CI

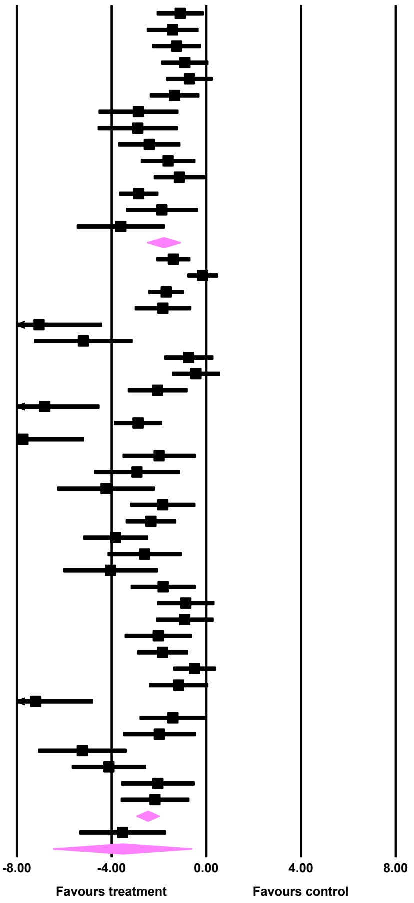

eFig4. Subgroup analysis by delivery routes for mNSS

Effects of stem cells on mNSS

Group by delivery routes	author	Std diff in means	Lower limit	Upper limit	p-Value
intra-arterial	Zhang-1,2006	-1.39	-2.08	-0.70	0.000
intra-arterial	Li F-1,2007	-1.83	-3.00	-0.66	0.002
intra-arterial	Li F-2,2007	-7.06	-9.70	-4.43	0.000
intra-arterial	Li F-3,2007	-5.19	-7.24	-3.14	0.000
intra-arterial	Li F-4,2007	-0.74	-1.75	0.27	0.152
intra-arterial	Li F-5,2007	-0.44	-1.43	0.55	0.388
intra-arterial	Seyfried,2008	-0.90	-1.87	0.07	0.068
intra-arterial		-1.91	-2.80	-1.01	0.000
intracerebral	Zhang-3,2006	-1.69	-2.41	-0.97	0.000
intracerebral	Liao,2009	-2.05	-3.29	-0.82	0.001
intracerebral	Otero,2010	-6.82	-9.11	-4.53	0.000
intracerebral	Tang,2010	-2.88	-3.87	-1.89	0.000
intracerebral	Feng-1,2011	-2.87	-4.52	-1.21	0.001
intracerebral	Feng-2,2011	-2.89	-4.56	-1.23	0.001
intracerebral	Otero,2011	-7.74	-10.30	-5.19	0.000
intracerebral	Yang CX,2011	-1.99	-3.51	-0.48	0.010
intracerebral	Chen J,2012	-2.93	-4.71	-1.14	0.001
intracerebral	Bao,2013	-2.34	-3.37	-1.30	0.000
intracerebral	Qin,2013	-3.83	-5.18	-2.48	0.000
intracerebral	Gao,2014	-2.60	-4.14	-1.07	0.001
intracerebral	Zhang,2015	-1.82	-3.16	-0.47	0.008
intracerebral	Xie-1,2016	-0.86	-2.05	0.32	0.152
intracerebral	Zhou,2016	-2.03	-3.42	-0.63	0.004
intracerebral	Ding,2017	-3.53	-5.34	-1.72	0.000
intracerebral	Zhang Y,2019	-1.17	-2.39	0.05	0.061
intracerebral	Chen X,2020	-7.20	-9.60	-4.80	0.000
intracerebral	Gao,2020	-3.61	-5.44	-1.77	0.000
intracerebral	Liu-1,2021	-1.41	-2.79	-0.02	0.046
intracerebral	Liu-2,2021	-1.98	-3.50	-0.47	0.010
intracerebral	Deng,2021	-2.04	-3.57	-0.51	0.009
intracerebral		-2.72	-3.25	-2.19	0.000
intraperitoneal	Hu-1,2021	-5.23	-7.08	-3.39	0.000
intraperitoneal	Hu-2,2021	-4.11	-5.66	-2.56	0.000
intraperitoneal		-4.63	-6.46	-2.80	0.000
intravenous	Seyfried-1,2006	-1.10	-2.07	-0.13	0.026
intravenous	Seyfried-2,2006	-1.42	-2.49	-0.36	0.009
intravenous	Seyfried-3,2006	-1.26	-2.27	-0.24	0.015
intravenous	Zhang-2,2006	-0.15	-0.77	0.47	0.630
intravenous	Seyfried-1,2010	-0.71	-1.67	0.24	0.143
intravenous	Seyfried-2,2010	-1.34	-2.36	-0.32	0.010
intravenous	Yang DM-1,2012	-2.41	-3.70	-1.12	0.000
intravenous	Yang DM-2,2012	-1.61	-2.74	-0.48	0.005
intravenous	Yang DM-3,2012	-1.13	-2.19	-0.08	0.035
intravenous	Wang SP,2012	-4.23	-6.27	-2.20	0.000
intravenous	Yang KL,2013	-1.83	-3.18	-0.49	0.008
intravenous	Wang CY,2015	-2.85	-3.66	-2.05	0.000
intravenous	Chen M,2015	-4.04	-6.02	-2.07	0.000
intravenous	Xie-2,2016	-0.91	-2.10	0.28	0.133
intravenous	Cui CM,2017	-1.87	-3.36	-0.39	0.014
intravenous	Zhang,2017	-1.85	-2.89	-0.80	0.001
intravenous	Kuramoto,2019	-0.50	-1.37	0.37	0.263
intravenous	Tang,2021	-2.17	-3.59	-0.74	0.003
intravenous		-1.61	-2.15	-1.07	0.000

Std diff in means and 95% CI

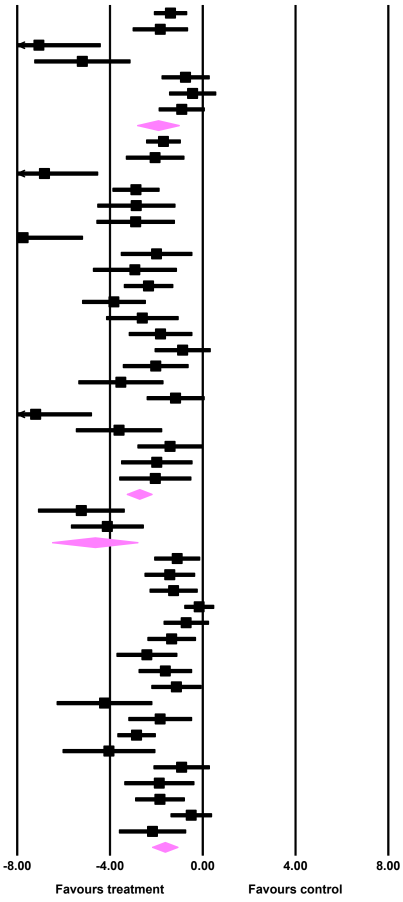

eFig5.Subgroup analysis by sources of stem cells for mNSS

Group by Sources of stem cells	author	Std diff in means	Lower limit	Upper limit	p-Value
allogeneic	Zhang-1,2006	-1.39	-2.08	-0.70	0.000
allogeneic	Zhang-2,2006	-0.15	-0.77	0.47	0.630
allogeneic	Zhang-3,2006	-1.69	-2.41	-0.97	0.000
allogeneic	Li F-1,2007	-1.83	-3.00	-0.66	0.002
allogeneic	Li F-2,2007	-7.06	-9.70	-4.43	0.000
allogeneic	Li F-3,2007	-5.19	-7.24	-3.14	0.000
allogeneic	Li F-4,2007	-0.74	-1.75	0.27	0.152
allogeneic	Li F-5,2007	-0.44	-1.43	0.55	0.388
allogeneic	Otero,2010	-6.82	-9.11	-4.53	0.000
allogeneic	Seyfried-1,2010	-0.71	-1.67	0.24	0.143
allogeneic	Seyfried-2,2010	-1.34	-2.36	-0.32	0.010
allogeneic	Tang,2010	-2.88	-3.87	-1.89	0.000
allogeneic	Otero,2011	-7.74	-10.30	-5.19	0.000
allogeneic	Yang CX,2011	-1.99	-3.51	-0.48	0.010
allogeneic	Chen J,2012	-2.93	-4.71	-1.14	0.001
allogeneic	Wang SP,2012	-4.23	-6.27	-2.20	0.000
allogeneic	Wang CY,2015	-2.85	-3.66	-2.05	0.000
allogeneic	Chen M,2015	-4.04	-6.02	-2.07	0.000
allogeneic	Cui CM,2017	-1.87	-3.36	-0.39	0.014
allogeneic	Ding,2017	-3.53	-5.34	-1.72	0.000
allogeneic	Zhang,2017	-1.85	-2.89	-0.80	0.001
allogeneic	Gao,2020	-3.61	-5.44	-1.77	0.000
allogeneic	Deng,2021	-2.04	-3.57	-0.51	0.009
allogeneic	Tang,2021	-2.17	-3.59	-0.74	0.003
allogeneic		-2.50	-3.05	-1.94	0.000
syngeneic	Gao,2014	-2.60	-4.14	-1.07	0.001
syngeneic	Zhang Y,2019	-1.17	-2.39	0.05	0.061
syngeneic	Chen X,2020	-7.20	-9.60	-4.80	0.000
syngeneic		-3.16	-4.81	-1.51	0.000
xenogeneic	Seyfried-1,2006	-1.10	-2.07	-0.13	0.026
xenogeneic	Seyfried-2,2006	-1.42	-2.49	-0.36	0.009
xenogeneic	Seyfried-3,2006	-1.26	-2.27	-0.24	0.015
xenogeneic	Seyfried,2008	-0.90	-1.87	0.07	0.068
xenogeneic	Liao,2009	-2.05	-3.29	-0.82	0.001
xenogeneic	Feng-1,2011	-2.87	-4.52	-1.21	0.001
xenogeneic	Feng-2,2011	-2.89	-4.56	-1.23	0.001
xenogeneic	Yang DM-1,2012	-2.41	-3.70	-1.12	0.000
xenogeneic	Yang DM-2,2012	-1.61	-2.74	-0.48	0.005
xenogeneic	Yang DM-3,2012	-1.13	-2.19	-0.08	0.035
xenogeneic	Yang KL,2013	-1.83	-3.18	-0.49	0.008
xenogeneic	Bao,2013	-2.34	-3.37	-1.30	0.000
xenogeneic	Qin,2013	-3.83	-5.18	-2.48	0.000
xenogeneic	Zhang,2015	-1.82	-3.16	-0.47	0.008
xenogeneic	Xie-1,2016	-0.86	-2.05	0.32	0.152
xenogeneic	Xie-2,2016	-0.91	-2.10	0.28	0.133
xenogeneic	Zhou,2016	-2.03	-3.42	-0.63	0.004
xenogeneic	Kuramoto,2019	-0.50	-1.37	0.37	0.263
xenogeneic	Liu-1,2021	-1.41	-2.79	-0.02	0.046
xenogeneic	Liu-2,2021	-1.98	-3.50	-0.47	0.010
xenogeneic	Hu-1,2021	-5.23	-7.08	-3.39	0.000
xenogeneic	Hu-2,2021	-4.11	-5.66	-2.56	0.000
xenogeneic		-1.94	-2.50	-1.38	0.000

Statistics for each study

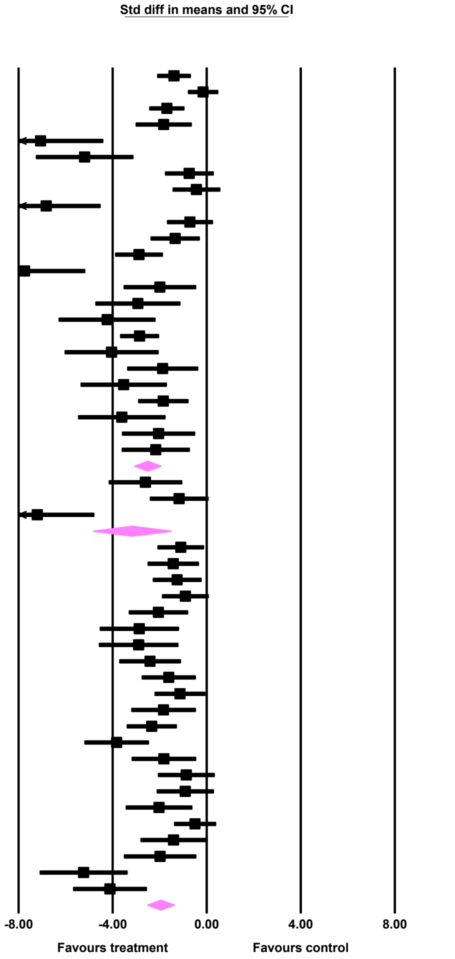

eFig6. Subgroup analysis by types of stem cells for mNSS

Group by types of stem cells	author	Std diff in means	Lower limit	Upper limit	p-Value
BM-EPCs	Zhang,2017	-1.85	-2.89	-0.80	0.001
BM-EPCs		-1.85	-4.36	0.67	0.150
IPSCs	Qin,2013	-3.83	-5.18	-2.48	0.000
IPSCs		-3.83	-6.48	-1.18	0.005
MSCs	Seyfried-1,2006	-1.10	-2.07	-0.13	0.026
MSCs	Seyfried-2,2006	-1.42	-2.49	-0.36	0.009
MSCs	Seyfried-3,2006	-1.26	-2.27	-0.24	0.015
MSCs	Zhang-1,2006	-1.39	-2.08	-0.70	0.000
MSCs	Zhang-2,2006	-0.15	-0.77	0.47	0.630
MSCs	Zhang-3,2006	-1.69	-2.41	-0.97	0.000
MSCs	Seyfried,2008	-0.90	-1.87	0.07	0.068
MSCs	Liao,2009	-2.05	-3.29	-0.82	0.001
MSCs	Otero,2010	-6.82	-9.11	-4.53	0.000
MSCs	Seyfried-1,2010	-0.71	-1.67	0.24	0.143
MSCs	Seyfried-2,2010	-1.34	-2.36	-0.32	0.010
MSCs	Feng-1,2011	-2.87	-4.52	-1.21	0.001
MSCs	Feng-2,2011	-2.89	-4.56	-1.23	0.001
MSCs	Otero,2011	-7.74	-10.30	-5.19	0.000
MSCs	Yang CX,2011	-1.99	-3.51	-0.48	0.010
MSCs	Yang DM-1,2012	-2.41	-3.70	-1.12	0.000
MSCs	Yang DM-2,2012	-1.61	-2.74	-0.48	0.005
MSCs	Yang DM-3,2012	-1.13	-2.19	-0.08	0.035
MSCs	Chen J,2012	-2.93	-4.71	-1.14	0.001
MSCs	Wang SP,2012	-4.23	-6.27	-2.20	0.000
MSCs	Yang KL,2013	-1.83	-3.18	-0.49	0.008
MSCs	Bao,2013	-2.34	-3.37	-1.30	0.000
MSCs	Wang CY,2015	-2.85	-3.66	-2.05	0.000
MSCs	Chen M,2015	-4.04	-6.02	-2.07	0.000
MSCs	Zhang,2015	-1.82	-3.16	-0.47	0.008
MSCs	Xie-1,2016	-0.86	-2.05	0.32	0.152
MSCs	Xie-2,2016	-0.91	-2.10	0.28	0.133
MSCs	Zhou,2016	-2.03	-3.42	-0.63	0.004
MSCs	Cui CM,2017	-1.87	-3.36	-0.39	0.014
MSCs	Ding,2017	-3.53	-5.34	-1.72	0.000
MSCs	Kuramoto,2019	-0.50	-1.37	0.37	0.263
MSCs	Zhang Y,2019	-1.17	-2.39	0.05	0.061
MSCs	Chen X,2020	-7.20	-9.60	-4.80	0.000
MSCs	Gao,2020	-3.61	-5.44	-1.77	0.000
MSCs	Liu-1,2021	-1.41	-2.79	-0.02	0.046
MSCs	Liu-2,2021	-1.98	-3.50	-0.47	0.010
MSCs	Hu-1,2021	-5.23	-7.08	-3.39	0.000
MSCs	Hu-2,2021	-4.11	-5.66	-2.56	0.000
MSCs	Deng,2021	-2.04	-3.57	-0.51	0.009
MSCs	Tang,2021	-2.17	-3.59	-0.74	0.003
MSCs		-2.20	-2.62	-1.78	0.000
NSCs	Li F-1,2007	-1.83	-3.00	-0.66	0.002
NSCs	Li F-2,2007	-7.06	-9.70	-4.43	0.000
NSCs	Li F-3,2007	-5.19	-7.24	-3.14	0.000
NSCs	Li F-4,2007	-0.74	-1.75	0.27	0.152
NSCs	Li F-5,2007	-0.44	-1.43	0.55	0.388
NSCs	Tang,2010	-2.88	-3.87	-1.89	0.000
NSCs	Gao,2014	-2.60	-4.14	-1.07	0.001
NSCs		-2.52	-3.54	-1.50	0.000

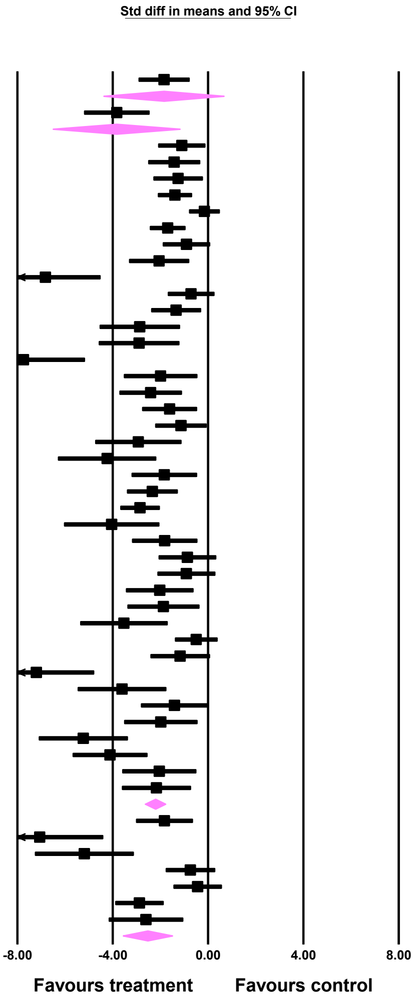

eFig7. Subgroup analysis by time administration of stem cells for mNSS

Effects of stem cells on mNSS

Group by time administration	author	Std diff in means	Lower limit	Upper limit	p-Value
《1 day	Seyfried-1,2006	-1.10	-2.07	-0.13	0.026
《1 day	Seyfried-2,2006	-1.42	-2.49	-0.36	0.009
《1 day	Seyfried-3,2006	-1.26	-2.27	-0.24	0.015
《1 day	Seyfried,2008	-0.90	-1.87	0.07	0.068
《1 day	Liao,2009	-2.05	-3.29	-0.82	0.001
《1 day	Seyfried-1,2010	-0.71	-1.67	0.24	0.143
《1 day	Seyfried-2,2010	-1.34	-2.36	-0.32	0.010
《1 day	Yang DM-1,2012	-2.41	-3.70	-1.12	0.000
《1 day	Wang SP,2012	-4.23	-6.27	-2.20	0.000
《1 day	Yang KL,2013	-1.83	-3.18	-0.49	0.008
《1 day	Bao,2013	-2.34	-3.37	-1.30	0.000
《1 day	Qin,2013	-3.83	-5.18	-2.48	0.000
《1 day	Gao,2014	-2.60	-4.14	-1.07	0.001
《1 day	Chen M,2015	-4.04	-6.02	-2.07	0.000
《1 day	Zhang,2015	-1.82	-3.16	-0.47	0.008
《1 day	Xie-1,2016	-0.86	-2.05	0.32	0.152
《1 day	Xie-2,2016	-0.91	-2.10	0.28	0.133
《1 day	Zhou,2016	-2.03	-3.42	-0.63	0.004
《1 day	Cui CM,2017	-1.87	-3.36	-0.39	0.014
《1 day	Ding,2017	-3.53	-5.34	-1.72	0.000
《1 day	Zhang,2017	-1.85	-2.89	-0.80	0.001
《1 day	Kuramoto,2019	-0.50	-1.37	0.37	0.263
《1 day	Chen X,2020	-7.20	-9.60	-4.80	0.000
《1 day	Gao,2020	-3.61	-5.44	-1.77	0.000
《1 day	Liu-1,2021	-1.41	-2.79	-0.02	0.046
《1 day	Liu-2,2021	-1.98	-3.50	-0.47	0.010
《1 day	Hu-1,2021	-5.23	-7.08	-3.39	0.000
《1 day	Tang,2021	-2.17	-3.59	-0.74	0.003
《1 day		-2.15	-2.66	-1.63	0.000
>1 day-1 week	Zhang-1,2006	-1.39	-2.08	-0.70	0.000
>1 day-1 week	Zhang-2,2006	-0.15	-0.77	0.47	0.630
>1 day-1 week	Zhang-3,2006	-1.69	-2.41	-0.97	0.000
>1 day-1 week	Li F-1,2007	-1.83	-3.00	-0.66	0.002
>1 day-1 week	Li F-2,2007	-7.06	-9.70	-4.43	0.000
>1 day-1 week	Otero,2010	-6.82	-9.11	-4.53	0.000
>1 day-1 week	Tang,2010	-2.88	-3.87	-1.89	0.000
>1 day-1 week	Feng-1,2011	-2.87	-4.52	-1.21	0.001
>1 day-1 week	Yang CX,2011	-1.99	-3.51	-0.48	0.010
>1 day-1 week	Yang DM-2,2012	-1.61	-2.74	-0.48	0.005
>1 day-1 week	Yang DM-3,2012	-1.13	-2.19	-0.08	0.035
>1 day-1 week	Chen J,2012	-2.93	-4.71	-1.14	0.001
>1 day-1 week	Zhang Y,2019	-1.17	-2.39	0.05	0.061
>1 day-1 week	Hu-2,2021	-4.11	-5.66	-2.56	0.000
>1 day-1 week	Deng,2021	-2.04	-3.57	-0.51	0.009
>1 day-1 week		-2.33	-3.03	-1.63	0.000
>1 week	Li F-3,2007	-5.19	-7.24	-3.14	0.000
>1 week	Li F-4,2007	-0.74	-1.75	0.27	0.152
>1 week	Li F-5,2007	-0.44	-1.43	0.55	0.388
>1 week	Feng-2,2011	-2.89	-4.56	-1.23	0.001
>1 week	Otero,2011	-7.74	-10.30	-5.19	0.000
>1 week		-2.78	-4.06	-1.51	0.000
NR	Wang CY,2015	-2.85	-3.66	-2.05	0.000
NR		-2.85	-5.33	-0.37	0.024

Statistics for each study

Std diff in means and 95% CI

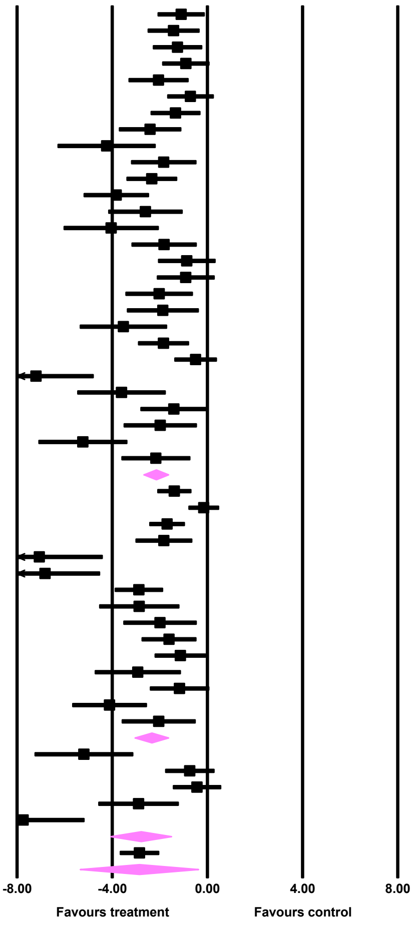

eFig8. Subgroup analysis by doses of stem cells for mNSS

Group by cell doses	author	Std diff in means	Lower limit	Upper limit	p-Value
[1-5]*10^6	Seyfried-1,2006	-1.10	-2.07	-0.13	0.026
[1-5]*10^6	Seyfried-2,2006	-1.42	-2.49	-0.36	0.009
[1-5]*10^6	Zhang-1,2006	-1.39	-2.08	-0.70	0.000
[1-5]*10^6	Zhang-2,2006	-0.15	-0.77	0.47	0.630
[1-5]*10^6	Zhang-3,2006	-1.69	-2.41	-0.97	0.000
[1-5]*10^6	Li F-1,2007	-1.83	-3.00	-0.66	0.002
[1-5]*10^6	Li F-2,2007	-7.06	-9.70	-4.43	0.000
[1-5]*10^6	Li F-3,2007	-5.19	-7.24	-3.14	0.000
[1-5]*10^6	Li F-4,2007	-0.74	-1.75	0.27	0.152
[1-5]*10^6	Li F-5,2007	-0.44	-1.43	0.55	0.388
[1-5]*10^6	Seyfried,2008	-0.90	-1.87	0.07	0.068
[1-5]*10^6	Otero,2010	-6.82	-9.11	-4.53	0.000
[1-5]*10^6	Seyfried-2,2010	-1.34	-2.36	-0.32	0.010
[1-5]*10^6	Feng-1,2011	-2.87	-4.52	-1.21	0.001
[1-5]*10^6	Feng-2,2011	-2.89	-4.56	-1.23	0.001
[1-5]*10^6	Otero,2011	-7.74	-10.30	-5.19	0.000
[1-5]*10^6	Yang DM-1,2012	-2.41	-3.70	-1.12	0.000
[1-5]*10^6	Yang DM-2,2012	-1.61	-2.74	-0.48	0.005
[1-5]*10^6	Yang DM-3,2012	-1.13	-2.19	-0.08	0.035
[1-5]*10^6	Wang SP,2012	-4.23	-6.27	-2.20	0.000
[1-5]*10^6	Yang KL,2013	-1.83	-3.18	-0.49	0.008
[1-5]*10^6	Qin,2013	-3.83	-5.18	-2.48	0.000
[1-5]*10^6	Wang CY,2015	-2.85	-3.66	-2.05	0.000
[1-5]*10^6	Chen M,2015	-4.04	-6.02	-2.07	0.000
[1-5]*10^6	Zhang,2015	-1.82	-3.16	-0.47	0.008
[1-5]*10^6	Xie-2,2016	-0.91	-2.10	0.28	0.133
[1-5]*10^6	Ding,2017	-3.53	-5.34	-1.72	0.000
[1-5]*10^6	Zhang,2017	-1.85	-2.89	-0.80	0.001
[1-5]*10^6	Kuramoto,2019	-0.50	-1.37	0.37	0.263
[1-5]*10^6	Chen X,2020	-7.20	-9.60	-4.80	0.000
[1-5]*10^6		-2.32	-2.81	-1.83	0.000
<1*10^6	Liao,2009	-2.05	-3.29	-0.82	0.001
<1*10^6	Seyfried-1,2010	-0.71	-1.67	0.24	0.143
<1*10^6	Tang,2010	-2.88	-3.87	-1.89	0.000
<1*10^6	Yang CX,2011	-1.99	-3.51	-0.48	0.010
<1*10^6	Chen J,2012	-2.93	-4.71	-1.14	0.001
<1*10^6	Bao,2013	-2.34	-3.37	-1.30	0.000
<1*10^6	Gao,2014	-2.60	-4.14	-1.07	0.001
<1*10^6	Xie-1,2016	-0.86	-2.05	0.32	0.152
<1*10^6	Zhou,2016	-2.03	-3.42	-0.63	0.004
<1*10^6	Zhang Y,2019	-1.17	-2.39	0.05	0.061
<1*10^6	Gao,2020	-3.61	-5.44	-1.77	0.000
<1*10^6	Liu-1,2021	-1.41	-2.79	-0.02	0.046
<1*10^6	Liu-2,2021	-1.98	-3.50	-0.47	0.010
<1*10^6	Hu-1,2021	-5.23	-7.08	-3.39	0.000
<1*10^6	Hu-2,2021	-4.11	-5.66	-2.56	0.000
<1*10^6	Deng,2021	-2.04	-3.57	-0.51	0.009
<1*10^6		-2.29	-2.97	-1.62	0.000
>5*10^6	Seyfried-3,2006	-1.26	-2.27	-0.24	0.015
>5*10^6	Tang,2021	-2.17	-3.59	-0.74	0.003
>5*10^6		-1.68	-3.52	0.17	0.075
NR	Cui CM,2017	-1.87	-3.36	-0.39	0.014
NR		-1.87	-4.62	0.87	0.181

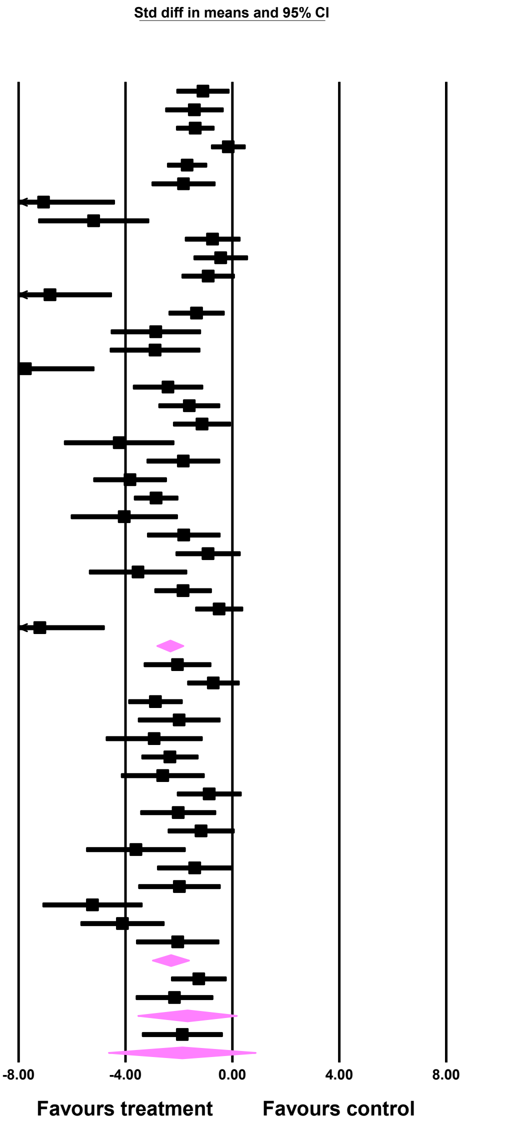

Supplement: Supplementary file 4 — Additional file 4. Funnel plot of sensitivity analysis for tissue loss. [file 13287_2022_3158_MOESM4_ESM.pdf]
