# Supplementary figures and images for: Efficacy of stem cell therapy in animal models of intracerebral hemorrhage: an updated meta-analysis
Source: Stem Cell Res Ther. 2022 Sep 5;13:452. doi: 10.1186/s13287-022-03158-7 (PMC9446670; doi:10.1186/s13287-022-03158-7)

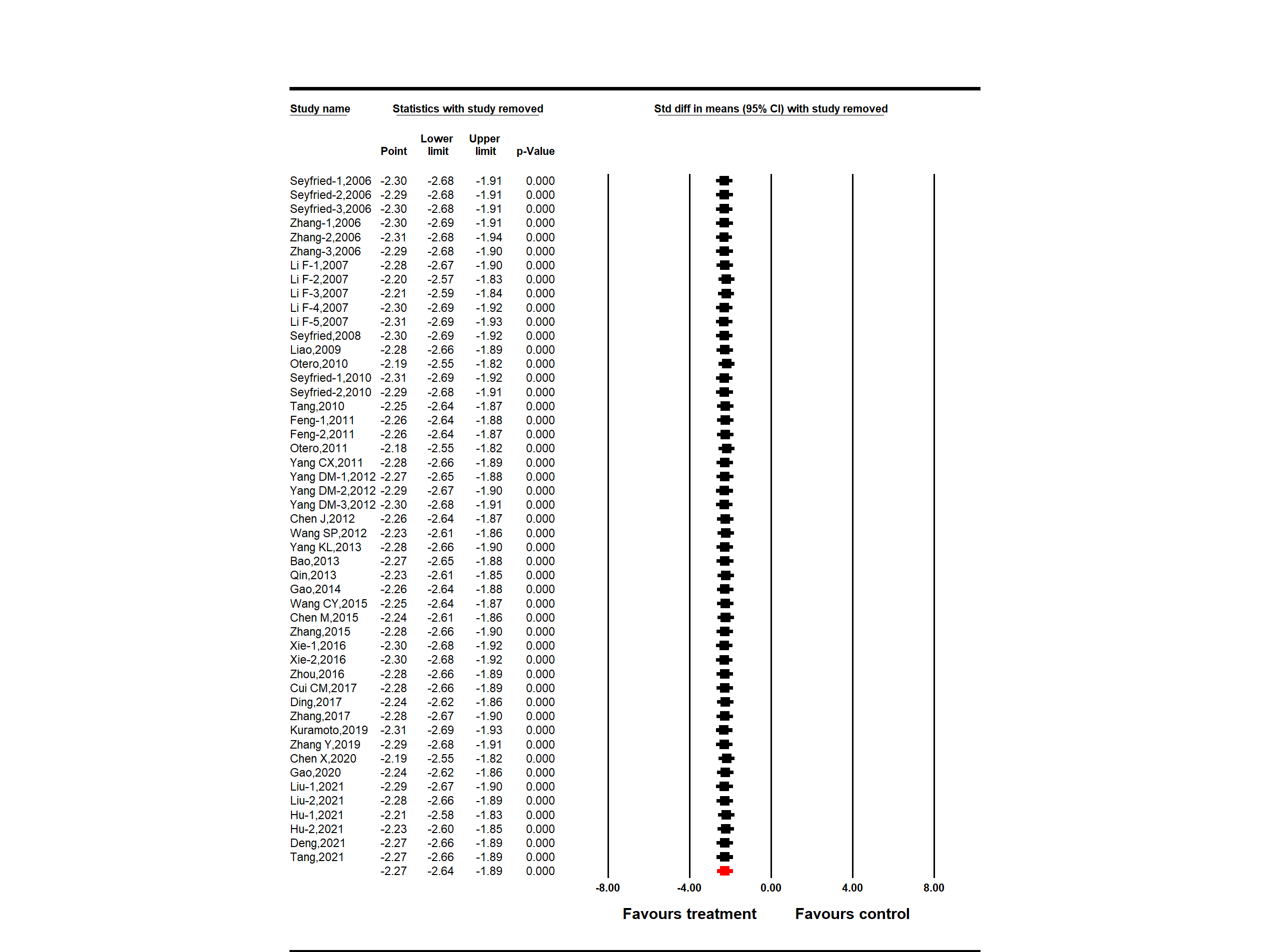

Supplement: Supplementary file 5 — Additional file 5. Funnel plot of sensitivity analysis for brain water content. [file 13287_2022_3158_MOESM5_ESM.tif]

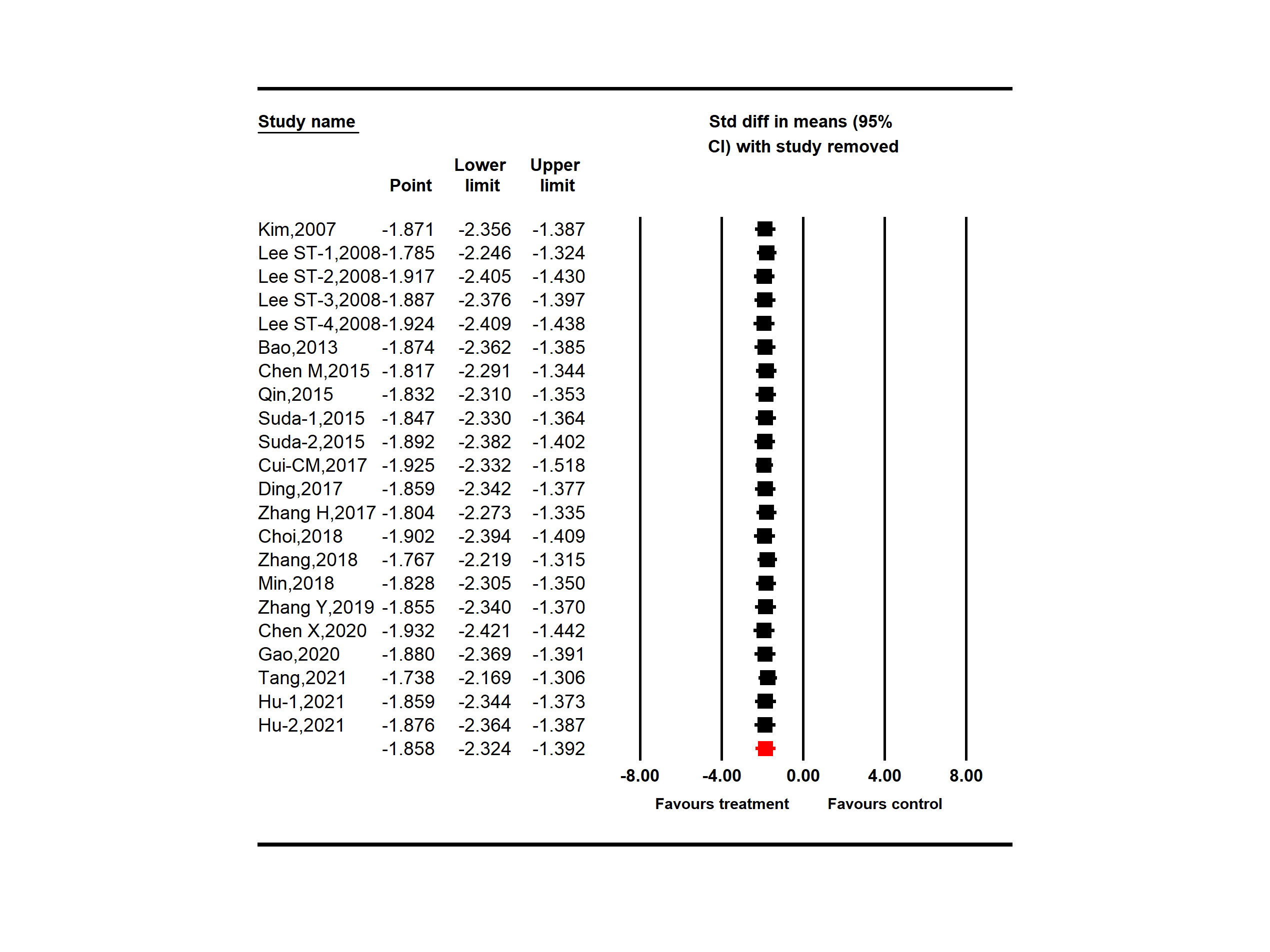

Supplement: Supplementary file 6 — Additional file 6. Subgroup analysis for mNSS. [file 13287_2022_3158_MOESM6_ESM.tif]

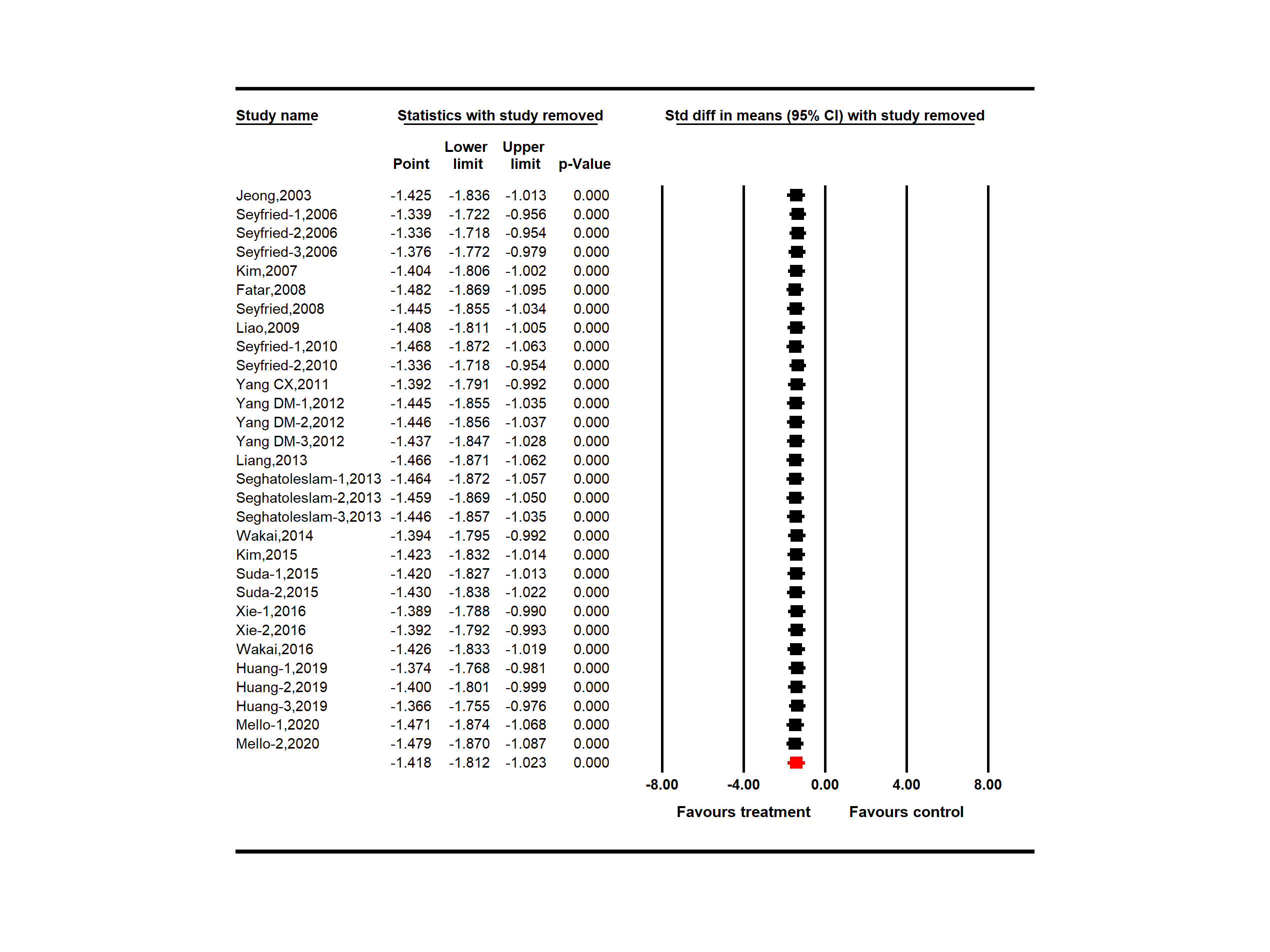

Supplement: Supplementary file 7 — Additional file 7. Subgroup analysis for tissue loss. [file 13287_2022_3158_MOESM7_ESM.tif]
